# Developmental Stage and Time Dictate the Fate of Wnt/β-Catenin-Responsive Stem Cells in the Mammary Gland

**DOI:** 10.1016/j.stem.2012.05.023

**Published:** 2012-08-02

**Authors:** Renée van Amerongen, Angela N. Bowman, Roel Nusse

**Affiliations:** 1 Department of Developmental Biology and Howard Hughes Medical Institute, Stanford University, Stanford, CA 94305, USA; 2 Present address: Netherlands Cancer Institute, 1066 CX Amsterdam, The Netherlands

## Abstract

The mammary epithelium undergoes extensive growth and remodeling during pregnancy, suggesting a role for stem cells. Yet their origin, identity, and behavior in the intact tissue remain unknown. Using an *Axin2*^*CreERT2*^ allele, we labeled and traced Wnt/β-catenin-responsive cells throughout mammary gland development. This reveals a switch in Wnt/β-catenin signaling around birth and shows that, depending on the developmental stage, *Axin2*^+^ cells contribute differently to basal and luminal epithelial cell lineages of the mammary epithelium. Moreover, an important difference exists between the developmental potential tested in transplantation assays and that displayed by the same cell population in situ. Finally, *Axin2*^+^ cells in the adult build alveolar structures during multiple pregnancies, demonstrating the existence of a Wnt/β-catenin-responsive adult stem cell. Our study uncovers dynamic changes in Wnt/β-catenin signaling in the mammary epithelium and offers insights into the developmental fate of mammary gland stem and progenitor cells.

## INTRODUCTION

The mammary gland harbors extraordinary proliferative and differentiation potential. This is illustrated by the rapid growth and extensive branching morphogenesis displayed during puberty, when the rudimentary ductal tree invades the surrounding stromal tissue to form the elaborate epithelial network that makes up the adult mammary gland parenchyma (Richert et al., 2000; Watson and Khaled, 2008). Even more striking is the proliferative capacity retained by the adult mammary epithelium, allowing dynamic tissue remodeling during pregnancy and lactation. This encompasses a massive expansion in cell number and the formation of milk-producing alveoli. Once lactation ceases, the alveolar structures regress in a process called involution, and the mammary gland returns to a prepregnancy-like state. Most remarkably, the cycle of pregnancy, lactation, and involution can repeat itself multiple times during the reproductive lifespan of an animal, suggesting that stem cells are present to ensure proper long-term maintenance of mammary tissue structure and function.

It is well accepted that stem cells do indeed exist in the adult mammary epithelium (reviewed by Visvader, 2009; Visvader and Smith, 2011). Over the last 50 years, the capacity to regenerate a ductal tree upon transplantation into the cleared fat pad has become the gold standard for analyzing stem cell potential (Daniel et al., 1968; Deome et al., 1959; Smith and Medina, 1988). The finding that cells with enhanced regenerative potential could be prospectively isolated by fluorescence-activated cell sorting (FACS) based on their Lin^−^;CD24^+^;CD29^hi^ (Shackleton et al., 2006) or Lin^−^;CD24^+^;CD49f^hi^ (Stingl et al., 2006) profile represented a major advance in the field. However, the regenerative potential tested in transplantation experiments does not necessarily reflect physiological behavior and cell fate in situ (Van Keymeulen et al., 2011). Consequently, true insight into stem cell origin and function in a normal developmental context can only be gained from lineage tracing analyses. For the mammary gland, this approach has long remained unexplored and key questions regarding the origin, identity, and behavior of mammary stem and progenitor cells remain.

Wnt/β-catenin signaling is instrumental for stem cell maintenance in multiple tissues and critical for mammary gland development and function. It is first required for mammary placode formation during embryogenesis (Chu et al., 2004; Veltmaat et al., 2004). In postnatal life, Wnt/β-catenin signaling controls branching morphogenesis and alveolar bud formation, as well as early lobuloalveolar development during pregnancy (Brisken et al., 2000; Kim et al., 2009; Lindvall et al., 2009; Macias et al., 2011; Teulière et al., 2005).

*Axin2* has been well established as a direct target gene of the Wnt/β-catenin pathway (Jho et al., 2002; Lustig et al., 2002). Moreover, we have previously demonstrated that *Axin2*^+^ cells have regenerative capacity in mammary gland transplantation experiments (Zeng and Nusse, 2010). Based on this, we used *Axin2* as a functional stem cell marker to study the contribution of Wnt/β-catenin-responsive cells to mammary gland development and differentiation using a lineage tracing approach. For this purpose, we have generated a mouse model, *Axin2*^*CreERT2*^, which allows us to mark these cells at different time points in situ and to track their behavior across distinct developmental stages.

## RESULTS

### *Axin2*^*CreERT2*^ Allows Lineage Tracing of Wnt/β-Catenin-Responsive Stem Cells

**Figure 1.**
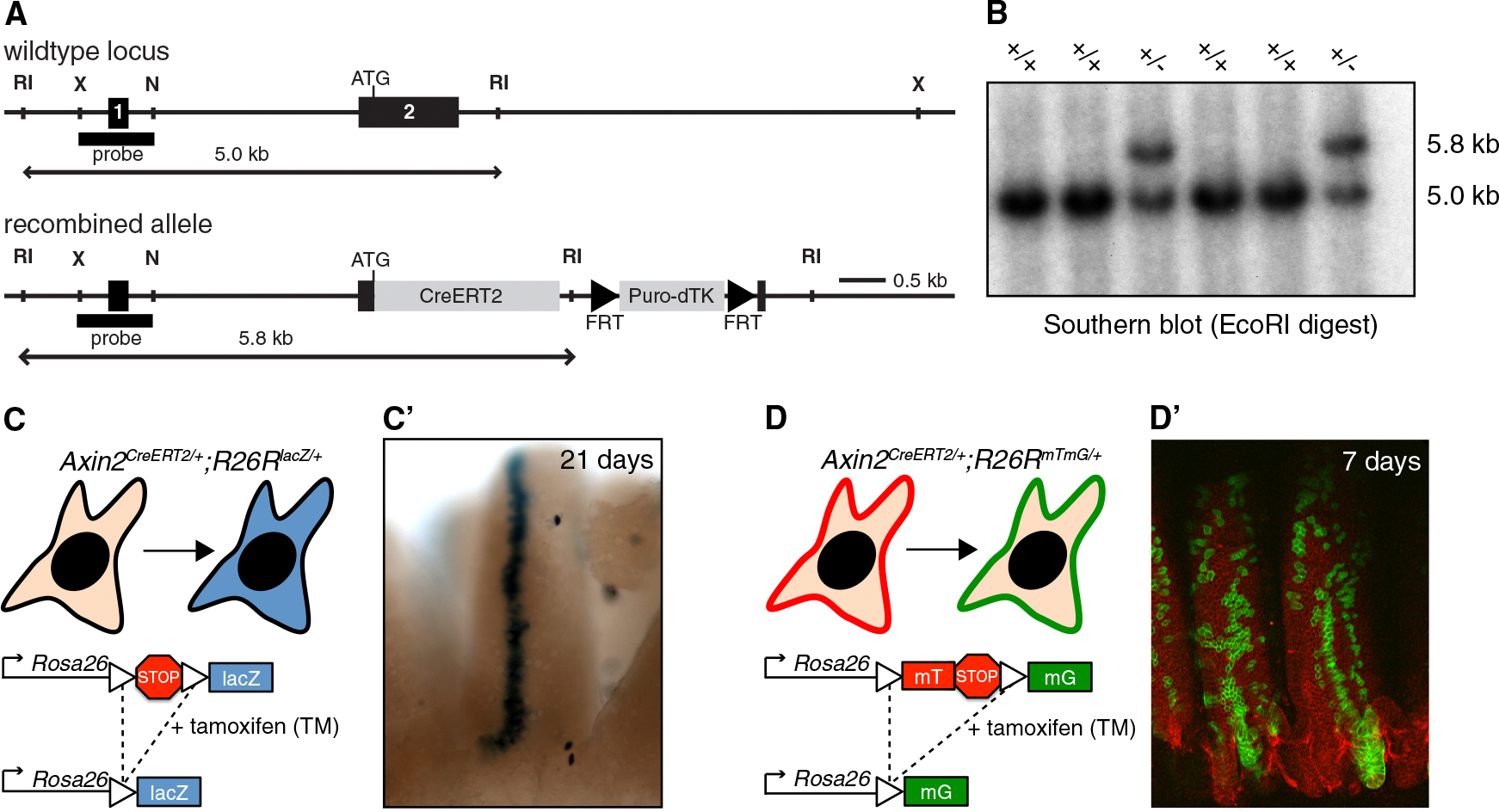
*Axin2*^*CreERT2*^ Marks Wnt/β-Catenin-Responsive Stem Cells (A) Targeting strategy to generate the *Axin2*^*CreERT2*^ knockin allele. Importantly, mice heterozygous for *Axin2* are phenotypically normal (Lustig et al., 2002; Zeng and Nusse, 2010) and cells with one copy of *Axin2* show the same responsiveness to Wnt-ligand stimulation as wild-type cells (Minear et al., 2010). (B) Southern blot analysis with a 5′ external probe of EcoRI-digested DNA from mouse embryonic stem cells, showing a 5.8 kb band in addition to the 5 kb wild-type band in clones that have undergone homologous recombination at the *Axin2* locus. (C, C′, D, and D′) Schematic of the lineage tracing strategy. *Axin2*^*CreERT2*^ mice are crossed to *R26R*^*lacZ*^ (C) or *R26R*^*mTmG*^ (D) reporter mice. A transient pulse of *Cre* activity induced by tamoxifen (TM) administration results in recombination of the floxed reporter locus, leaving behind a permanent genetic mark. Cells gain expression of lacZ (C) or switch from red (membrane-bound dTomato, mT) to green (membrane-bound GFP, mG) (D). (C′ and D′) *Axin2*^*CreERT2*^ marks intestinal stem cells. Blue (C′, 21 days post-TM) or green (D′, 7 days post-TM) ribbons representing tracts of labeled cells descendent from individual recombined stem cells can be traced from the bottom of the crypt to the top of the villus. See also Figure S1.

We generated an *Axin2*^*CreERT2*^ allele by knocking a tamoxifen-inducible *Cre* recombinase (*Cre*^*ERT2*^) into the endogenous *Axin2* gene (Figure 1A). *Axin2*^*CreERT2*/+^ mice derived from embryonic stem cells that had undergone homologous recombination at the *Axin2* locus (Figure 1B) were crossed to the *Rosa26-lacZ* (*R26R*^*lacZ*^) and *Rosa26-mT/mG* (*R26R*^*mTmG*^) reporter strains (Muzumdar et al., 2007; Soriano, 1999). Upon administration of tamoxifen (TM), *Cre* activity in *Axin2*^*CreERT2*/+^;*R26R*^*lacZ*/+^ or *Axin2*^*CreERT2*/+^;*R26R*^*mTmG*/+^ mice results in sporadic recombination of the floxed reporter loci, leaving behind a permanent, genetic mark in the form of persistent lacZ expression (Figure 1C; in *R26R*^*lacZ*/+^) or a switch from membrane-bound dTomato to membrane-bound green fluorescent protein (GFP) expression (Figure 1D; in *R26R*^*mTmG*/+^). Not only does the recombined cell carry this label for the remainder of its lifespan, it also passes the expression of lacZ or GFP on to its offspring, thereby allowing the developmental fate of the Wnt/β-catenin-responsive lineage to be traced.

As a proof of principle, we tested the ability of *Axin2*^*CreERT2*^ to mark stem cells in the intestinal epithelium. This tissue turns over every 3–7 days, and Wnt/β-catenin-responsive stem cells at the bottom of the intestinal crypt are critical for its maintenance (Korinek et al., 1998; Muncan et al., 2006; Van der Flier et al., 2007). It was previously demonstrated that after Cre-mediated recombination of a floxed reporter allele, these stem cells give rise to entirely labeled crypt/villus units that persist for long periods of time (Barker et al., 2007). In agreement with these published data, we find that *Axin2*^*CreERT2*^ initially marks cells at the crypt base (see Figures S1A–S1D available online). Within a week, the offspring of these cells have migrated up along the crypt/villus axis, resulting in ribbons of labeled cells that run from the bottom of the crypt to the tip of the villus (Figures 1C′ and 1D′). Recombined cells persist up to 350 days (Figures S1E–S1H and data not shown), which is well beyond the lifespan of the intestinal epithelium, thus demonstrating that *Axin2*^*CreERT2*^ marks intestinal stem cells.

### Wnt/β-Catenin Signaling in the Mammary Placode Marks the Prospective Luminal Lineage

**Figure 2.**
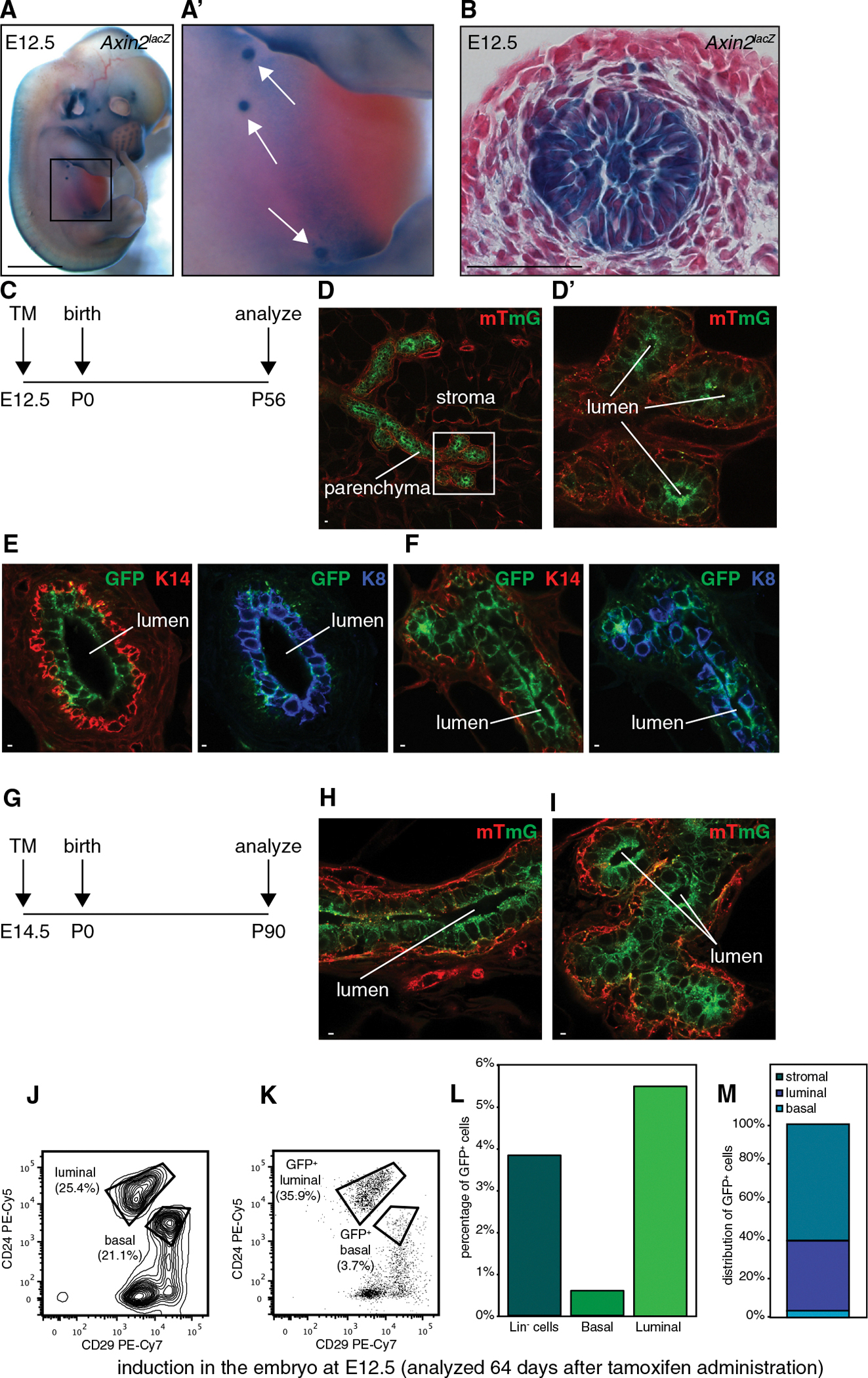
Wnt/β-Catenin-Responsive Cells in the Embryo Contribute to the Luminal Cell Lineage (A and A′) Whole-mount X-gal staining demonstrating *Axin2*^*lacZ*^ expression in mammary placodes of an E12.5 embryo (A). A close-up of the boxed area is shown in (A′). Scale bar represents 2 mm. (B) Histological tissue section of an X-gal-stained mammary placode at E12.5 showing strong *Axin2*^*lacZ*^ expression in the mammary bud, but not the overlying epithelium or surrounding mesenchyme. Scale bar represents 100 μm. (C) Experimental schedule used in (D)–(F) to analyze the contribution of embryonic Wnt/β-catenin-responsive cells to the adult mammary gland. (D and D′) Whole-mount confocal microscopy, allowing simultaneous detection of recombined GFP^+^ and unrecombined dTomato^+^ cells in *Axin2*^*CreERT2*/+^;*R26R*^*mTmG*/+^ mice (D). The GFP^+^ offspring of *Axin2*^+^ cells in the E12.5 embryo have become incorporated in the luminal cell layer of the adult mammary gland parenchyma. The image depicts a distal branch with a close-up of the ductal end buds (boxed area) shown in (D′). Scale bar represents 10 μm. (E and F) Immunostaining of proximal (E) and distal (F) portions of the ductal epithelium with basal (K14, red) and luminal (K8, blue) markers confirms that GFP^+^ cells are restricted to the luminal cell lineage. Scale bars represent 2 μm. (G) Experimental schedule used in (H) and (I) to analyze the contribution of embryonic *Axin2*^+^ cells to the adult mammary gland. (H and I) Whole-mount confocal microscopy showing that the GFP^+^ offspring of *Axin2*^+^ cells in the E14.5 embryo have become incorporated in the luminal cell layer of the adult mammary gland parenchyma in the ducts (H) and distal end buds (I). Scale bars represent 2 μm. (J–M) FACS analysis (see Figure S2 for details on the procedure) on the mammary gland from a littermate of the animal shown in (D)–(F). (J) FACS plot showing discrimination of basal (Lin^−^;CD24^+^;CD29^hi^) and luminal (Lin^−^;CD24^hi^; CD29^+^) cell populations. (K) FACS plot showing distribution of GFP^+^ cells across the different cell populations. (L) Bar graph showing contribution of GFP^+^ cells to Lin^−^ (4.0% GFP^+^ cells), basal (0.7% GFP^+^ cells), and luminal (5.7% GFP^+^ cells) cell populations. (M) Bar graph showing relative distribution of GFP^+^ cells among stromal, luminal, and basal epithelial cell populations.

The earliest signs of mammary placode formation are the localized expression of Wnt10b and corresponding activity of Wnt/β-catenin reporter gene expression (Al Alam et al., 2011; Chu et al., 2004; Veltmaat et al., 2004). Indeed, we detected robust expression of *Axin2*^*lacZ*^ in mammary placodes of embryonic day (E) 12.5 embryos (Figures 2A and 2B). To study the contribution of these Wnt/β-catenin-responsive cells to mammary gland development, we administered TM to pregnant females at E12.5 (Figures 2C–2F), E14.5 (Figures 2G–2I), or E17.5 (data not shown) and analyzed the mammary glands from female offspring once these mice had reached adulthood.

Using whole-mount confocal fluorescence microscopy, we identified large, GFP^+^ cell clones (0–2 per gland) in the otherwise dTomato^+^ mammary epithelium of *Axin2*^*CreERT2*/+^;*R26R*^*mTmG*/+^ mice. These clones extended to the most distal tips of the branched ductal network (Figures 2D and 2I). Within these clones, GFP^+^ cells were restricted to the luminal cell fate, as confirmed by costaining with basal (K14) and luminal (K8) markers as well as by FACS analysis (Figures 2E, 2F, and 1J–2M; n = 5 mice analyzed in total). A similar picture was observed regardless of whether recombination was induced at E12.5, E14.5, or E17.5. Thus, we conclude that Wnt/β-catenin signaling in the embryonic mammary bud marks the prospective luminal cell lineage and that even within the mammary placode, some cells with elevated *Axin2* expression are already luminal specified.

### Wnt/β-Catenin-Responsive Stem Cells in the Prepubescent Mammary Gland Are Restricted to the Basal Lineage

**Figure 3.**
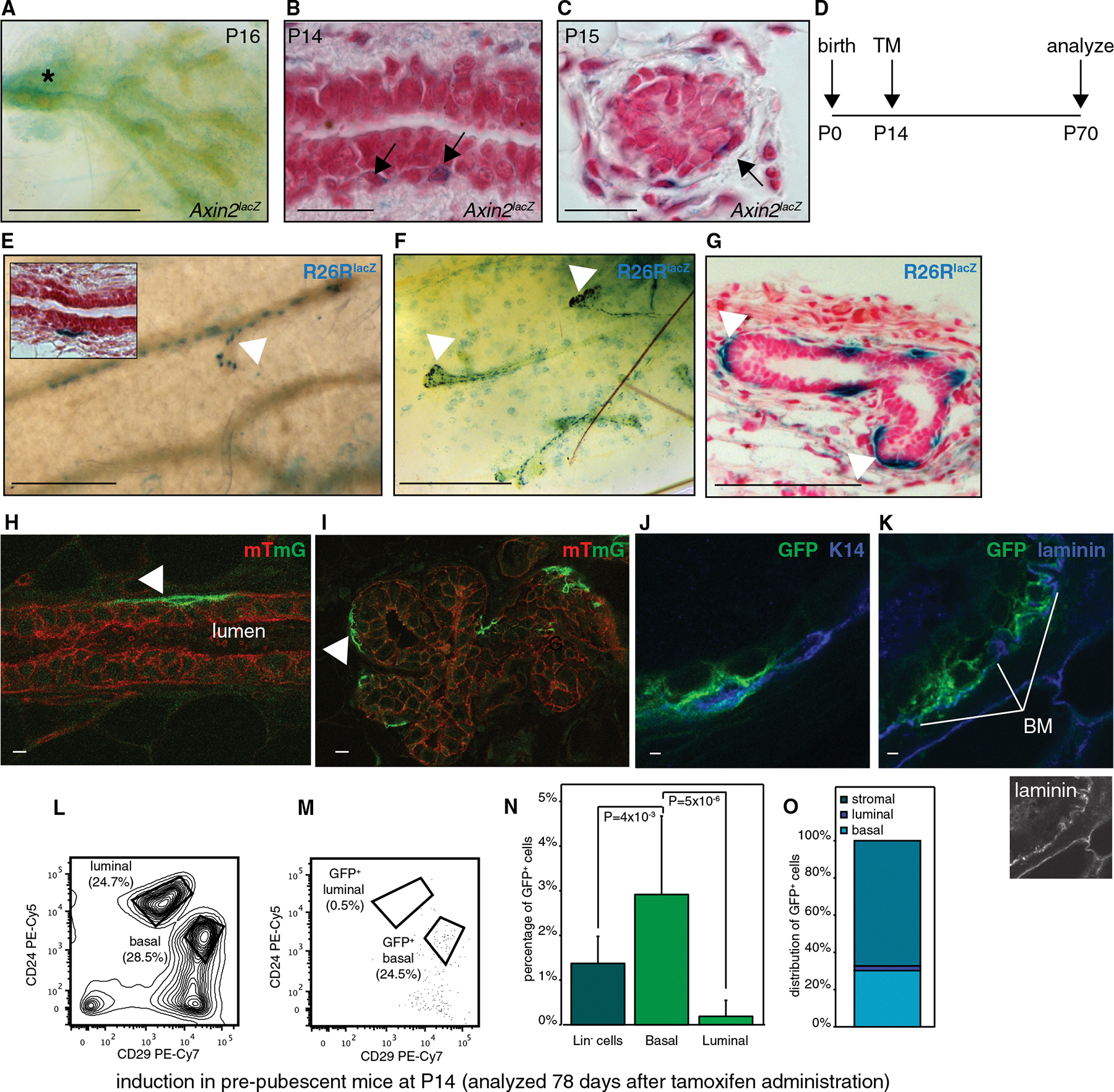
Wnt/β-Catenin-Responsive Cells in the Prepubescent Mammary Gland Are Restricted to the Basal Lineage (A–C) Whole-mount preparation (A) and histological tissue sections (B and C) of X-gal-stained mammary glands from 2-week-old *Axin2*^*lacZ*^ mice, showing diffuse reporter activity in the stroma surrounding the rudimentary tree (asterisk in A) and in rare cells associated with primary (B) and secondary (C) ducts (arrows). Scale bar represents 0.5 mm in (A) and 50 μm in (B) and (C). (D) Experimental schedule used in (E)–(O) to analyze the contribution of prepubescent Wnt/β-catenin-responsive cells to the adult mammary gland. (E) Whole-mount image of an X-gal-stained *Axin2*^*CreERT2*/+^;*R26R*^*lacZ*/+^ mammary gland, showing tracts of labeled cells (arrowhead). Inset depicts a histological tissue section of the same sample, demonstrating that labeled cells reside in the basal, but not the luminal, layer. Scale bar represents 500 μm. See also Figure S3. (F and G) Whole-mount image (F) and corresponding histological tissue section (G) of an X-gal-stained *Axin2*^*CreERT2*/+^;*R26R*^*lacZ*/+^ mammary gland, demonstrating that labeled cells (arrowheads) are found along the entire length of the epithelial network, including the most distal tips. Scale bar represents 1 mm in (F) and 100 μm in (G). (H and I) Whole-mount confocal images of an *Axin2*^*CreERT2*/+^;*R26R*^*mTmG*/+^ mammary gland, confirming that prepubescent Wnt/β-catenin-responsive cells traced into adulthood are incorporated in the basal layer as GFP^+^ cells (arrowheads) along the entire length of the epithelial network (H), including the most distal tips (I). Scale bar represents 10 μm. (J and K) Immunostaining for K14 (J) and laminin (K) demonstrates that GFP^+^ cells express basal cell markers and lie on top of the basement membrane (BM). Scale bar represents 1 μm. (L–O) FACS analysis on adult virgin *Axin2*^*CreERT2*/+^;*R26R*^*mTmG*/+^ mammary glands after administration of TM between P14 and P16. (L) FACS plot showing discrimination of basal (Lin^−^;CD24^+^;CD29^hi^) and luminal (Lin^−^;CD24^hi^;CD29^+^) cell populations. (M) FACS plot showing distribution of GFP^+^ cells across the different cell populations. (N) Bar graph showing contribution of GFP^+^ cells to Lin^−^, basal, and luminal cell populations. (O) Bar graph showing relative distribution of GFP^+^ cells among stromal, luminal, and basal epithelial cell populations, confirming that Wnt/β-catenin signaling marks the basal, rather than the luminal, cell lineage in prepubescent mice. Graphs in (N) and (O) show pooled data from n = 14 adult *Axin2*^*CreERT2*/+^;*R26R*^*mTmG*/+^ mice, analyzed at 9–13 weeks of age after a 7–11 week trace period. p values demonstrate statistical differences between the indicated cell populations (t test). Error bars indicate SD.

Relatively little is known about the role of Wnt/β-catenin signaling in early postnatal development, but *Axin2*^*lacZ*^ reporter activity can be detected in rare cells associated with primary and secondary ducts of the rudimentary mammary epithelium in 2-week-old mice (Figures 3A–3C). To probe the contribution of these *Axin2*^+^ cells to the expanding epithelial network, we injected prepubescent (postnatal days 14–16 [P14–P16]) mice with TM. Mice were then allowed to develop through puberty into adulthood, at which point mammary glands were analyzed for the presence of labeled cells (Figure 3D). A single dose of TM resulted in only a few lacZ^+^ or GFP^+^ cell clusters in *Axin2CreERT2*/+;*R26RlacZ*/+ or *Axin2CreERT2*/+;*R26RmTmG*/+ animals (Figures 3E–3K and Figure S3), demonstrating that the descendants of Wnt/β-catenin-responsive cells in the prepubescent mammary gland undergo massive proliferation during puberty and become incorporated into the basal layer of the adult mammary epithelium. Remarkably, lacZ^+^ or GFP^+^ cells were deposited along the entire length of the ductal network, including the most distal tips (Figures 3F and 3I). This suggests that labeled cells retain their basal cell fate even while migrating at the leading edge of the epithelial ducts during puberty.

FACS analysis confirmed the basal cell fate of the prepubescent Wnt/β-catenin-responsive cell lineage (Figures 3L–3O). In all cases, GFP^+^ cells made up a larger proportion of the Lin^−^; CD24^+^;CD29^hi^ basal (Figure 3N; 2.9% ± 1.8%, n = 14 animals) than of the Lin^−^;CD24^hi^;CD29^+^ luminal cell population (Figure 3N; 0.2% ± 0.3%, n = 14 animals), with not a single GFP^+^ cell being detected in the luminal population in 7/14 animals. However, the majority of GFP^+^ cells (Figure 3O; 67.2% ± 14.2%, 1,257 out of 1,964 GFP^+^ events) probably represent stromal cells, as they fell into neither the gated basal (Figure 3O; 30.8% ± 13.8%, 659 out of 1,964 GFP^+^ events) nor the gated luminal (Figure 3O; 2.1% ± 3.7%, 48 out of 1,964 GFP^+^ events) cell population.

Taken together, the embryonic (Figure 2) and prepuberty (Figure 3) tracing experiments suggest that a previously unrecognized switch in Wnt/β-catenin signaling activity takes place around birth. As a result, *Axin2* expression marks the prospective luminal lineage between E12.5 and E17.5 but the basal lineage between P14 and P16.

### *Axin2*^+^ Cells in the Prepubescent Mammary Gland Are Unipotent Stem Cells

**Figure 4.**
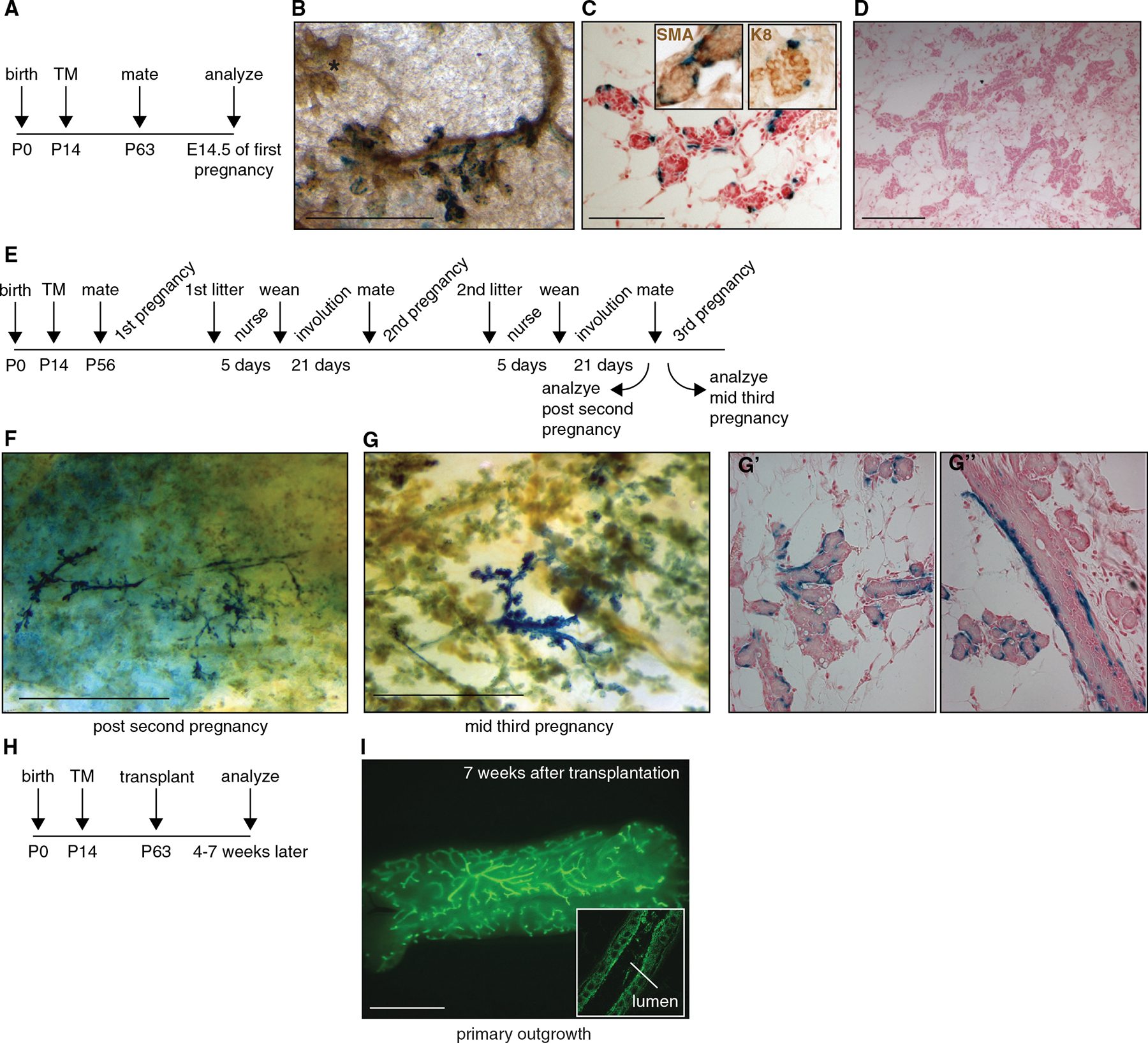
Transplantation of Lineage-Restricted Wnt/β-Catenin-Responsive Stem Cells Unlocks a Multipotent Regenerative Potential (A) Experimental schedule used in panels (B)–(D) for analyzing the contribution of the prepubescent Wnt/β-catenin-responsive lineage to the formation of alveoli. (B) Whole-mount image of an X-gal-stained *Axin2*^*CreERT2*/+^;*R26R*^*lacZ*/+^ mammary gland at day 14.5 of gestation, showing clusters of labeled cells surrounding growing alveolar structures. Asterisk in the top left corner indicates a nearby, unlabeled alveolar cluster. Scale bar represents 500 μm. (C) Histological tissue section of the same gland as in (B), demonstrating that labeled cells are confined to the basal layer. They stain positive for the myoepithelial marker SMA (left inset) and surround the luminal alveolar cells, which stain positive for K8 (right inset). Scale bar represents 100 μm. (D) Histological tissue section from the same gland as in (B) and (C), demonstrating that most of the epithelium does not contain labeled cells. Scale bar represents 200 μm. (E) Experimental schedule used in panels (F) and (G) to track the fate of prepubescent *Axin2*^+^ cells across multiple rounds of pregnancy. *Axin2*^*CreERT2*/+^;*R26R*^*lacZ*/+^ pups received a single dose of TM around P14. Mice were allowed to develop into adulthood, were mated at P56, and were monitored for signs of pregnancy. Mice were allowed to nurse their pups to ensure complete terminal differentiation of the mammary gland. After forced weaning on postnatal day 5, mice were housed for 21 days to allow complete involution of the epithelium. Mice were then remated and submitted to up to two additional cycles of pregnancy, lactation, and involution according to an identical schedule. Nulliparous siblings that received TM simultaneously were never mated and were analyzed together with their multiparous littermates as controls (data not shown). (F and G) Whole-mount preparations of X-gal-stained *Axin2*^*CreERT2*/+^;*R26R*^*lacZ*/+^ mammary glands isolated upon completing 21 days of involution after the second pregnancy (F) or midgestation at day 14.5 of the third pregnancy (G), showing dense clusters of lacZ^+^ basal cells among an otherwise unlabeled mammary parenchyma, in secondary and tertiary branches as well as surrounding the alveoli. (G′ and G″) Tissue sections of the whole-mount preparation shown in (G), demonstrating that the offspring of Wnt/β-catenin-responsive cells in the prepubescent mammary gland survive multiple rounds of pregnancy and continue to give rise to basal, but not luminal, cells with each subsequent gestation in both ducts and alveolar structures. (H) Experimental setup to test the capacity of the prepubescent Wnt/β-catenin-responsive lineage to regenerate a ductal tree. (I) Whole-mount image of a primary outgrowth derived from the transplantation of 140 GFP^+^ Lin^−^;CD24^+^;CD29^hi^ cells isolated from adult virgin *Axin2*^*CreERT2*/+^; *R26R*^*mTmG*/+^ mice in which recombination was induced at 14 to 16 days of age. Scale bar represents 500 μm. Inset: whole-mount confocal image of a regenerated ductal tree, demonstrating the presence of GFP^+^ basal and luminal layers. Scale bar represents 10 μm. See also Table S1.

We next sought to test the fate of the prepubescent Wnt/β-catenin-responsive cell lineage during pregnancy (Figure 4A). After recombination at P14 and analysis of midpregnant animals, we observed labeled cells that retained the appearance of basal, myoepithelial cells around developing alveoli (Figures 4B and 4C). Importantly, the majority of the gland did not contain labeled cells in the epithelium (Figure 4D), indicating that labeled cell clusters surrounding the alveoli are the clonal offspring of a single recombination event (Buckingham and Meilhac, 2011). Thus, the Wnt/β-catenin-responsive lineage marked in the prepubescent mammary gland undergoes proliferation as the epithelial network expands during puberty and pregnancy but remains restricted to the basal cell fate.

Upon weaning of the offspring, the mammary gland undergoes massive tissue remodeling. The alveolar structures regress as a result of widespread apoptosis and clearance of milk-secreting cells. To determine whether the Wnt/β-catenin-responsive cells in the prepubescent mammary gland classify as stem cells, we tested whether they survived multiple rounds of tissue turnover. By tracing their fate across multiple pregnancies (Figures 4E–4G), we found that these cells were long lived and continued to proliferate and increase in number with each subsequent gestation. This resulted in large areas, comprising multiple alveolar clusters, being cupped by the clonal offspring of a recombined cell in an otherwise unlabeled mammary epithelium (Figures 4F and 4G). Within these clusters, however, labeled cells were still restricted to the basal layer (Figures 4G′ and 4G″). From this, we conclude that Wnt/β-catenin-responsive cells in the basal layer of the prepubescent mammary gland are unipotent stem cells that retain long-term proliferative potential.

### Lineage-Restricted Wnt/β-Catenin-Responsive Stem Cells Behave as Multipotent Stem Cells when Transplanted

Given that the descendants of prepubescent *Axin2*^+^ cells did not adopt luminal cell fates in lineage tracing experiments, we hypothesized that they would also be unlikely to give rise to a new ductal tree after transplantation into the cleared fat pad. To test this, we prepared mammary cell suspensions from 9- to 13-week-old *Axin2*^*CreERT2*/+^;*R26R*^*mTmG*/+^ mice that had received TM between P14 and P16. GFP^+^ cells were sorted from the basal Lin^−^;CD24^+^;CD29^hi^ population by flow cytometry, after which their regenerative potential was analyzed in transplantation assays.

Surprisingly, these cells were highly efficient at regenerating a new ductal tree. As few as 50 cells were able to give rise to GFP^+^ outgrowths with normal basal and luminal layers, which was fully capable of developing alveoli during pregnancy (Figures 4H and 4I, Table S1, and data not shown). Moreover, when we reisolated the (now entirely GFP^+^) Lin^−^;CD24^+^;CD29^hi^ basal cells from these primary outgrowths, they were also able to regenerate a mammary gland in secondary transplantation experiments (data not shown). Thus, we conclude that transplantation into the cleared fat pad unlocks a regenerative potential that is not utilized during normal developmental conditions.

### *Axin2*^+^ Cells Build the Mammary Epithelial Network during Puberty

Predominant outgrowth of the mammary epithelium occurs during puberty, when branching morphogenesis and elongation of the ductal tree are driven by specialized, rapidly dividing terminal end bud (TEB) structures. By the time the mouse reaches adulthood, the epithelial network has invaded the entire stromal fat pad and the TEBs regress. Multiple Wnt-pathway genes, including ligands and receptors, are expressed by either the TEB itself or by the surrounding stroma (Kouros-Mehr and Werb, 2006), suggesting that Wnt/β-catenin-responsive cells contribute to outgrowth of the mammary epithelial network during puberty.

**Figure 5.**
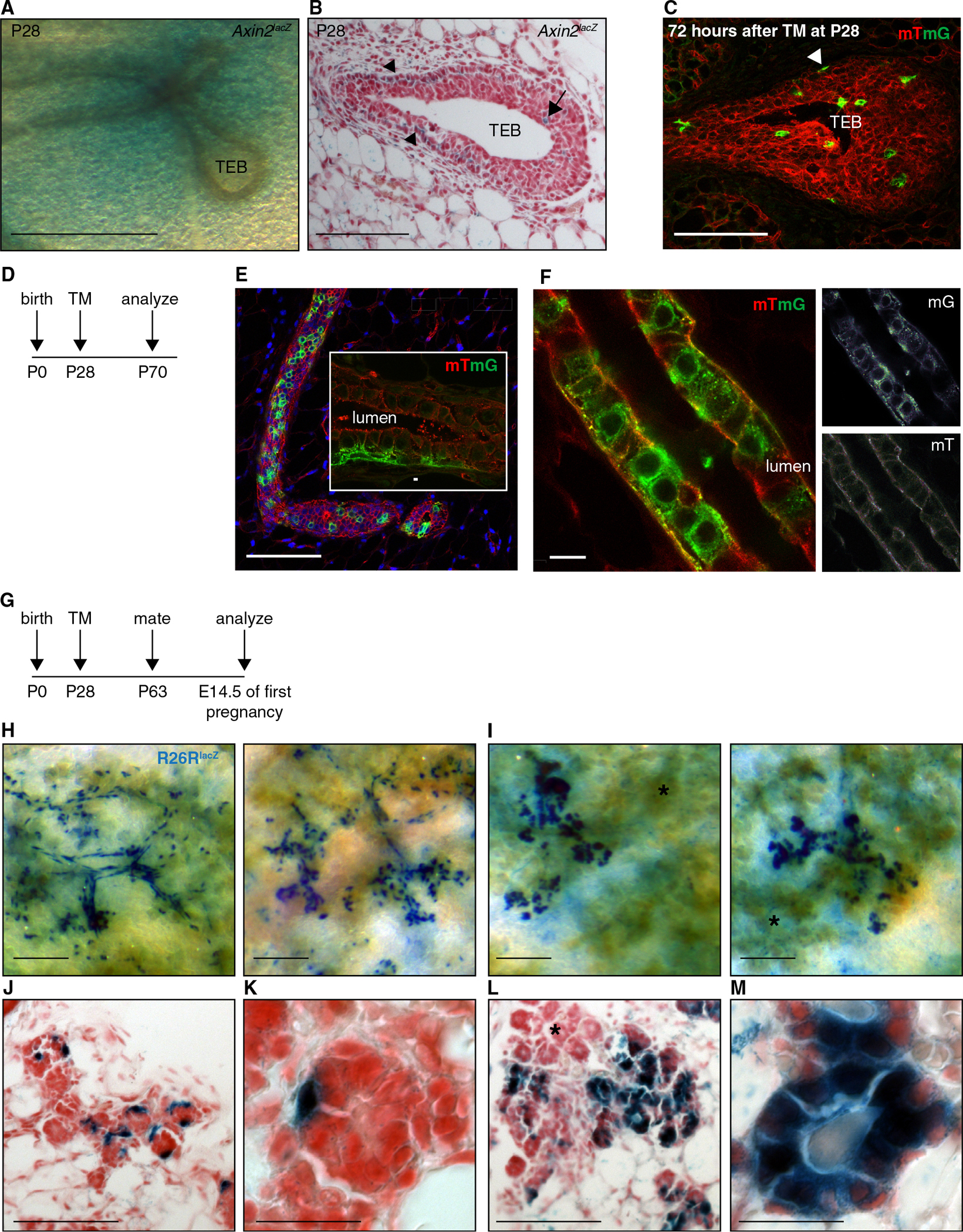
Both Basal and Luminal Precursors Are Wnt/β-Catenin-Responsive during Puberty (A and B) Whole-mount preparation (A) and histological tissue section (B) of an X-gal-stained pubescent *Axin2*^*lacZ*^ mammary gland, showing expression in the stroma (A) surrounding the terminal end buds (TEBs) as well as in epithelial cells (B) of the TEB body (arrow) and basal layer (arrowheads). Scale bar represents 500 μm in (A) and 100 μm in (B). (C) Whole-mount confocal microscopy of a pubescent *Axin2*^*CreERT2*/+^;*R26R*^*mTmG*/+^ mammary gland 72 hr after TM administration, demonstrating recombination in the basal layer (arrowhead) and body cells of the TEB. Scale bar represents 100 μm. (D) Experimental setup used in panels (E) and (F). (E and F) Histological tissue sections of adult virgin *Axin2*^*CreERT2*/+^;*R26R*^*mTmG*/+^ mammary glands in which recombination was induced during puberty. Tracts of GFP^+^ cells have become incorporated into the mature ductal network, where they have adopted both basal (inset in E) and luminal (F) cell fates. Scale bar represents 100 μm in (E), 2 μm in the inset in (E), and 10 μm in (F). (G) Experimental setup used in panels (H)–(M). (H–M) Whole-mount preparations (H and I) and tissue sections (J–M) of X-gal-stained *Axin2*^*CreERT2*/+^;*R26R*^*lacZ*/+^ mammary glands, isolated from a 12-week-old pregnant mouse at day 14.5 of gestation after TM administration at P28, demonstrating clonal outgrowth of basal (H, J, and K) or luminal (I, L, and M) cell clusters in alveolar structures. Note that labeled clones reside among a majority of unlabeled alveoli (examples indicated with asterisks in I and L). Scale bars represent 500 μm in (H) and (I), 100 μm in (J) and (L), and 20 μm in (K) and (M).

To test this hypothesis, we first analyzed the expression pattern of *Axin2*^*lacZ*^ reporter mice at P28, when puberty has commenced and the TEBs have formed. In whole-mount preparations of X-gal-stained mammary glands, *Axin2*^*lacZ*^ expression was most prominently detected in a zone of stroma surrounding the neck region of the TEBs (Figure 5A). However, in histological tissue sections, *Axin2*^*lacZ*^ expression was also apparent in the epithelium itself, both in rare TEB body cells (arrow in Figure 5B) and in basal cells more proximal to the TEB (arrowheads in Figure 5B). A similar picture was observed in pubescent *Axin2*^*CreERT2*^ mice that were analyzed 2–3 days after TM administration in conjunction with either the *R26R*^*lacZ*^ (data not shown) or the *R26R*^*mTmG*^ reporter (Figure 5C).

Next, we tracked the developmental fate of these Wnt/β-catenin-responsive cells by administering a single dose of TM to pubescent *Axin2*^*CreERT2*/+^;*R26R*^*mTmG*/+^ mice and analyzing the contribution of labeled cells to the mature epithelial network once the mice had reached adulthood (Figure 5D). In 10- to 12-week-old virgins, the offspring of Wnt/β-catenin-responsive cells could be visualized as GFP^+^ tracts (Figures 5E and 5F) that had become incorporated into both basal (inset in Figure 5E) and luminal (Figure 5F) layers of the ductal epithelium. During pregnancy, the offspring of *Axin2*^+^ cells that were labeled at puberty underwent massive clonal expansion, forming distinct clusters of either basal or luminal alveolar cells (Figures 5H–5M). Taken together, these data demonstrate that during puberty, Wnt/β-catenin-responsive cells give rise to both basal and luminal cell lineages of the expanding epithelial network. However, given the appearance of independent basal and luminal cell clones during pregnancy, the bilayered ductal epithelium is likely to arise from independent basal and luminal precursors, both of which are *Axin2*^+^ during puberty.

### *Axin2*^*CreERT2*^ Marks Basal Cells in the Adult Virgin Mammary Gland

By initiating lineage tracing experiments in embryos, prepubescent, or pubescent mice, we were able to establish that *Axin2*^+^ cells contribute differently to basal and luminal cell lineages of the mammary gland depending on the developmental stage of the tissue. We next sought to resolve the role of Wnt/β-catenin signaling in the adult mammary gland, where the hierarchy and origin of the stem cells that build alveoli during pregnancy remain ill understood.

**Figure 6.**
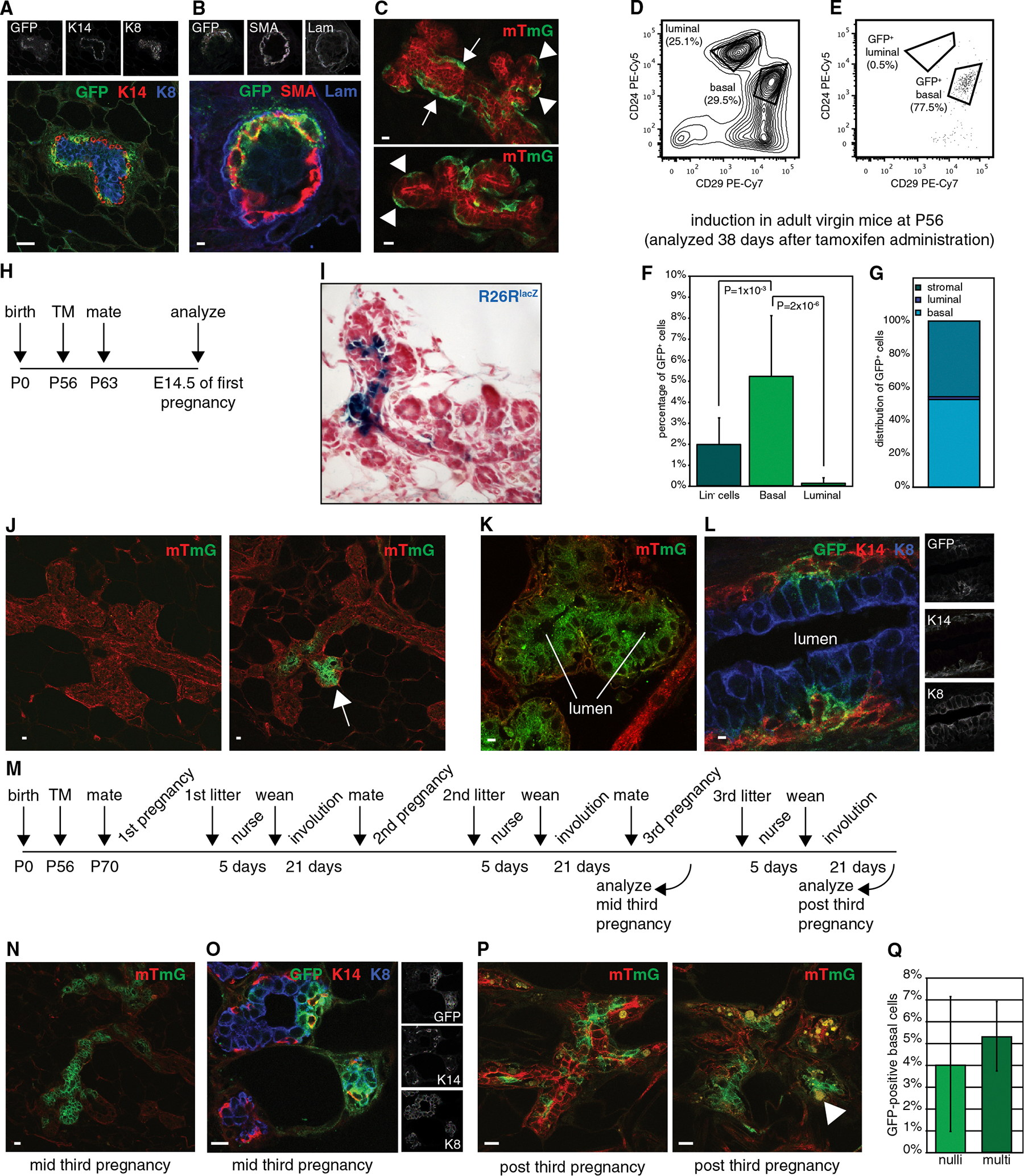
Wnt/β-Catenin-Responsive Cells Are Adult Mammary Gland Stem Cells that Build Alveoli during Multiple Rounds of Pregnancy (A and B) Immunofluorescent detection of GFP^+^ cells 48 hr after administration of TM to 9-week-old adult virgin *Axin2*^*CreERT2*/+^;*R26R*^*mTmG*/+^ mice. Labeled cells reside in the basal layer, marked by K14 (A) or SMA (B) expression. They are not found in the luminal layer, marked by K8 (A) expression, and lie on top of the basement membrane, marked by laminin (B). Scale bar represents 10 μm in (A) and 2 μm in (B). (C) Whole-mount confocal microscopy of an adult virgin *Axin2*^*CreERT2*/+^;*R26R*^*mTmG*/+^ mammary gland (analyzed 8 days after receiving the final of four consecutive injections administered 48 hr apart and totaling 8 mg/25 g of TM), showing recombined GFP^+^ cells in the context of an otherwise unrecombined dTomato^+^ epithelium. Labeled cells line the ductal epithelium (arrows in top panel) and surround alveolar (arrowheads in top panel) as well as ductal end buds (arrowheads in bottom panel). Scale bars represent 10 μm. (D–G) FACS analysis on *Axin2*^*CreERT2*/+^;*R26R*^*mTmG*/+^ adult virgin mammary glands after TM administration between P56 and P68. (D) FACS plot showing discrimination of basal (Lin^−^;CD24^+^;CD29^hi^) and luminal (Lin^−^;CD24^hi^;CD29^+^) cell populations. (E) FACS plot showing distribution of GFP^+^ cells across the different mammary cell populations. (F) Bar graph showing contribution of GFP^+^ cells to the Lin^−^, basal, and luminal cell populations. (G) Bar graph showing the relative distribution of GFP^+^ cells among stromal, luminal, and basal epithelial cell populations, demonstrating that in adult mice Wnt/β-catenin signaling marks the basal, rather than the luminal, cell lineage. Graphs in (F) and (G) show pooled data from n = 13 adult *Axin2*^*CreERT2*/+^;*R26R*^*mTmG*/+^ mice with a trace period ranging from 48 hr to 66 days. p values demonstrate statistical differences between the indicated cell populations (t test). Error bars indicate SD. See also Figure S2. (H) Experimental setup used in panels (I)–(L). (I and J) X-gal-stained tissue section (I) and whole-mount confocal microscopy (J) of *Axin2*^*CreERT2*/+^;*R26R*^*lacZ*/+^ (I) and *Axin2*^*CreERT2*/+^;*R26R*^*mTmG*/+^ (J) mammary glands at day 14.5 of gestation after TM administration to adult virgin mice. The majority of alveolar clusters do not contain lacZ^+^ or GFP^+^ cells, underscoring that recombination has been a sporadic event. (J) Some *Axin2*^*CreERT2*/+^;*R26R*^*mTmG*/+^ alveoli (right) contain dense areas of GFP^+^ cells (arrow), representing the clonal offspring of a single recombination event. (K) Close-up of an alveolar structure, demonstrating that Wnt/β-catenin-responsive cells in the adult virgin mammary gland have contributed to luminal alveolar cells. (L) Immunostaining of a nonalveolar part of an *Axin2*^*CreERT2*/+^;*R26R*^*mTmG*/+^ mammary gland at E14.5 of gestation. GFP^+^ cells remain localized to the basal layer of the ductal epithelium and express the basal marker K14, but not the luminal marker K8. Scale bars represent 10 μm in (J) and 2 μm in (K) and (L). (M) Experimental setup used in panels (N)–(Q). See Figure S7 for details. (N) Whole-mount confocal image of an *Axin2*^*CreERT2*/+^;*R26R*^*mTmG*/+^ mammary gland at day 14.5 of the third pregnancy. Labeled cells still generate dense areas of GFP^+^ alveoli. (O) Immunostaining of alveoli containing GFP^+^ cells during the third round of pregnancy. Adjacent labeled cells expressing basal (K14) and luminal (K8) markers can be identified. See Figure S7 for close-up. (P) Whole-mount confocal images of *Axin2*^*CreERT2*/+^;*R26R*^*mTmG*/+^ mammary glands isolated after 21 days of involution after the third pregnancy. GFP^+^ cells are still being cleared from regressing alveoli (white arrowhead). Yellow signal comes from autofluorescent infiltrating and/or dying cells. Scale bars represent 10 μm in (N)–(P). (Q) Quantification of labeled cells reveals no difference in the percentage of GFP^+^ basal (Lin^−^;CD24^+^;CD29^hi^) cells between nulliparous mice (n = 3) and multiparous littermates (n = 3) that have gone through three complete cycles of pregnancy and involution. See also Figures S4–S7.

*Axin2*^*lacZ*^ is expressed by a small proportion of basal cells in the adult mammary epithelium (Zeng and Nusse, 2010). Using confocal microscopy, we imaged the glands from 9-week-old virgin *Axin2*^*CreERT2*/+^;*R26R*^*mTmG*/+^ mice that had been injected with TM 48 hr prior. Analysis of GFP-expression in conjunction with basal and luminal markers confirmed that recombined cells resided in the basal layer (Figures 6A and 6B).

When we administered a high dose of TM to adult virgin mice and analyzed the mammary glands by whole-mount confocal microscopy 2 weeks after recombination, we also observed labeled cells with the appearance of elongated myoepithelial cells along the primary and secondary ducts (arrows in Figure 6C). In addition, GFP^+^ cells surrounded the lateral, or alveolar, buds (arrowheads in top panel of Figure 6C), which go on to form tertiary branches and secretory alveoli during pregnancy. Finally, labeled cells cradled the end buds at the distal tip of the epithelium (arrowheads in bottom panel of Figure 6C).

We next analyzed the mammary glands from a total of 13 animals, all of which received TM as adult virgins between the ages of 8–10 weeks, by flow cytometry (Figures 6D–6G). Irrespective of the length of the trace (ranging from 48 hr to 66 days after TM administration), we consistently observed a higher percentage of GFP^+^ cells in the Lin^−^;CD24^+^;CD29^hi^ basal cell population (Figure 6F; 5.2% ± 2.9%, n = 13 animals) than in the bulk of Lin^−^ cells (Figure 6F; 2.0% ± 1.2%, n = 13 animals). Conversely, GFP^+^ cells in the Lin^−^;CD24^hi^;CD29^+^ luminal cell population were rare (Figure 6F; 0.2% ± 0.2%, n = 13 animals), with not a single GFP^+^ cell being detected in the luminal population in 5/13 animals. On average, basal cells made up approximately half of the total number of labeled cells (Figure 6G; 53.9% ± 16.7%, 1,176 out of 1,969 GFP^+^ events). Only a small fraction (Figure 6G; 1.5% ± 1.9%, 23 out of 1,969 GFP^+^ events) of the GFP^+^ cells fell into the luminal cell population. The remainder (Figure 6G; 44.6% ± 17.3%) mostly express low levels of CD24 (Figure 6E) and are likely to be stromal fibroblasts, which also express *Axin2* as determined by histological analyses (data not shown). Taken together, these analyses demonstrate that in the adult, *Axin2*^*CreERT2*^ preferentially marks cells in the basal Lin^−^;CD24^+^;CD29^hi^ mammary epithelial cell population, corroborating our previous results obtained with *Axin2*^*lacZ*^ reporter mice. Moreover, cells marked by *Axin2*^*CreERT2*^ remain localized to the basal layer of the adult virgin mammary epithelium as long as the tissue remains quiescent.

### Clonal Expansion of Labeled Wnt/β-Catenin-Responsive Cells in Alveolar Structures during Pregnancy

Transplantation assays with GFP^+^ Lin^−^;CD24^+^;CD29^hi^ cells marked by *Axin2*^*CreERT2*^ in the adult virgin demonstrated that these cells were able to regenerate an entire epithelial network with great efficiency (summarized in Table S1 and Figure S4). However, both our own (Figures 4H and 4I) and recently published data (Van Keymeulen et al., 2011) demonstrate that transplantation into the cleared fat pad does not reflect the normal developmental potential of a mammary gland stem cell in its natural environment. We therefore tested the fate of these cells during pregnancy. Little remains known about how adult mammary stem cells function in vivo to build the specialized alveolar structures. In particular, the existence of a bipotent adult stem cell that could give rise to both basal and luminal alveolar cells remains contested.

To track the fate of *Axin2*^+^ cells in the adult, we injected 8-week-old virgins with a single dose of TM and, starting 1 week after recombination, performed timed matings to induce pregnancy (Figure 6H). At day 14.5 of gestation, the vast majority of the mammary epithelium was lacZ^−^ or GFP^−^, confirming that TM administration had resulted in sporadic recombination of the floxed reporter alleles. A few regions, however, harbored dense areas of labeled cells (Figures 6I and 6J and Figure S5). Whereas GFP^+^ cells remained restricted to the basal layer along the main ducts, alveoli also contained GFP^+^ luminal alveolar cells, which differentiate to produce and secrete milk toward the end of pregnancy (Figures 6K and 6L). Of note, within these GFP^+^ clusters, we were able to detect adjacent GFP^+^ luminal (K8-positive) and basal (K14-positive) cells (Figures S5 and S6).

Thus, lineage tracing demonstrates that *Axin2*^+^ cells in the adult virgin mammary epithelium contribute to both basal and luminal cells in the alveoli that arise during the first pregnancy. Moreover, individual lacZ^+^ or GFP^+^ cell clusters are separated by large stretches of unlabeled epithelium, suggesting that they are the clonal offspring of a single recombination event (Buckingham and Meilhac, 2011).

### Wnt/β-Catenin-Responsive Cells in the Adult Virgin Mammary Gland Are Long Lived Stem Cells that Give Rise to Alveoli during Multiple Pregnancies

To track the fate of adult *Axin2*^+^ cells across multiple rounds of pregnancy, we administered TM to adult virgin mice. These were then allowed to complete up to three cycles of pregnancy, lactation, and involution, after which mammary glands were analyzed for the presence of labeled cells.

After two complete cycles of pregnancy (~15–16 weeks after TM administration) mammary glands from multiparous *Axin2*^*CreERT2*/+^;*R26R*^*lacZ*/+^ mice were easily discernable from their nulliparous counterparts (Figures S7A–S7C). Although involution is generally considered to be complete after approximately 2 weeks (Richert et al., 2000; Strange et al., 1992), even after 21 days of involution, the mammary glands from multiparous animals had a far more disordered appearance than those of nulliparous littermates. The epithelial network was denser, with an obvious increase in tertiary branches and remnants of alveoli (arrows in Figures S7B and S7C). Labeled cells were found to persist in both nulliparous and multiparous animals. In nulliparous mice, they could sometimes be seen as tracts of lacZ^+^ cells that ran along the epithelial duct (arrowhead in Figure S7B). In other cases, both nulliparous and multiparous littermates contained dense clusters of labeled cells in a subset of the smaller branches (arrowheads in Figure S7C), potentially reflecting the generation of new side branches that sprout from existing ducts with recurrent estrous cycles (Andres and Strange, 1999; Brisken, 2002). Multiparous *Axin2*^*CreERT2*/+^;*R26R*^*lacZ*/+^ mice also contained lacZ^+^ regions of regressing alveoli (Figure S7D).

A similar picture was observed in *Axin2*^*CreERT2*/+^;*R26R*^*mTmG*/+^ mice, in which labeled cells still contributed to the building of alveolar clusters during the third pregnancy (Figure 6N). Within these clusters, we were again able to detect adjacent GFP^+^ basal (K14-positive) and luminal (K8-positive) cells (Figure 6O and Figures S7E–S7M). Finally, labeled cells remained present 21 days after weaning of the third litter (~22 weeks after TM administration), when remnants of alveoli were still being cleared (Figure 6P). Upon analyzing the distribution of GFP^+^ cells in the different mammary cell populations, we observed large variation in the amount of GFP^+^ luminal Lin^−^;CD24^hi^;CD29^+^ cells among mice that had been traced for more than 20 weeks, irrespective of their parity status (data not shown). However, the percentage of GFP^+^ basal Lin^−^;CD24^+^;CD29^hi^ cells was comparable between nulliparous littermates (4.1% ± 3.1%, n = 3 animals) and mice that had completed three cycles of pregnancy and involution (5.3% ± 1.6%, n = 3 animals; Figure 6Q). Thus, *Axin2*^+^ cells in the basal layer of the adult mammary gland are able to self-renew.

Because Wnt/β-catenin-responsive cells in the adult virgin mammary gland survive the complete turnover of the lobuloalveolar compartment as defined by the dynamic remodeling of the mammary epithelium that occurs postpregnancy, we conclude that *Axin2*^*CreERT2*^ marks long-lived adult mammary gland stem cells. These cells form the building blocks of alveolar structures during multiple rounds of pregnancy. The fact that labeled basal and luminal cells can be detected side by side within the same cell cluster during multiple pregnancies suggests a clonal relationship between the two and implies that one bipotent Wnt/β-catenin-responsive cell can generate an entire alveolar structure.

## DISCUSSION

Much effort has been put into delineating the relationships between different epithelial cell populations in the mammary gland. This has resulted in two models, each of which proposes a hierarchy of stem and progenitor cells in the mammary epithelium (Visvader, 2009; Visvader and Smith, 2011). The first of these models assumes the existence of independent ductal and lobular progenitors, both of which have the capacity to give rise to basal as well as luminal offspring. This model is mostly based on (serial) transplantation experiments with limiting amounts of cells, which sometimes results in lobule-limited or duct-limited outgrowths (Bruno and Smith, 2011; Kamiya et al., 1998; Kordon and Smith, 1998; Smith, 1996). A second model instead proposes an early separation between the basal and luminal cell lineages. Mainly based on in vitro colony formation assays (Stingl et al., 1998), it predicts, among others, the existence of a myoepithelial progenitor. This model is supported by recent lineage tracing experiments, which demonstrated that during puberty both the myoepithelial and the luminal lineage contain long-lived, unipotent stem cells (Van Keymeulen et al., 2011).

### Dynamic Changes in Wnt/β-Catenin Signaling during Mammary Gland Development

Wnt/β-catenin signaling controls various aspects of mammary gland development and differentiation during both embryogenesis and postnatal life and is critical for stem cell maintenance and function in multiple tissues. Thus, *Axin2* is both a direct transcriptional target of the Wnt/β-catenin pathway (Lustig et al., 2002) and a defined stem cell marker. We show that *Axin2* is expressed by only a subset of epithelial cells in the postnatal mammary gland epithelium. By labeling these cells at different developmental time points, our study reveals important conceptual points regarding the origin and behavior of mammary gland stem cells.

**Figure 7.**
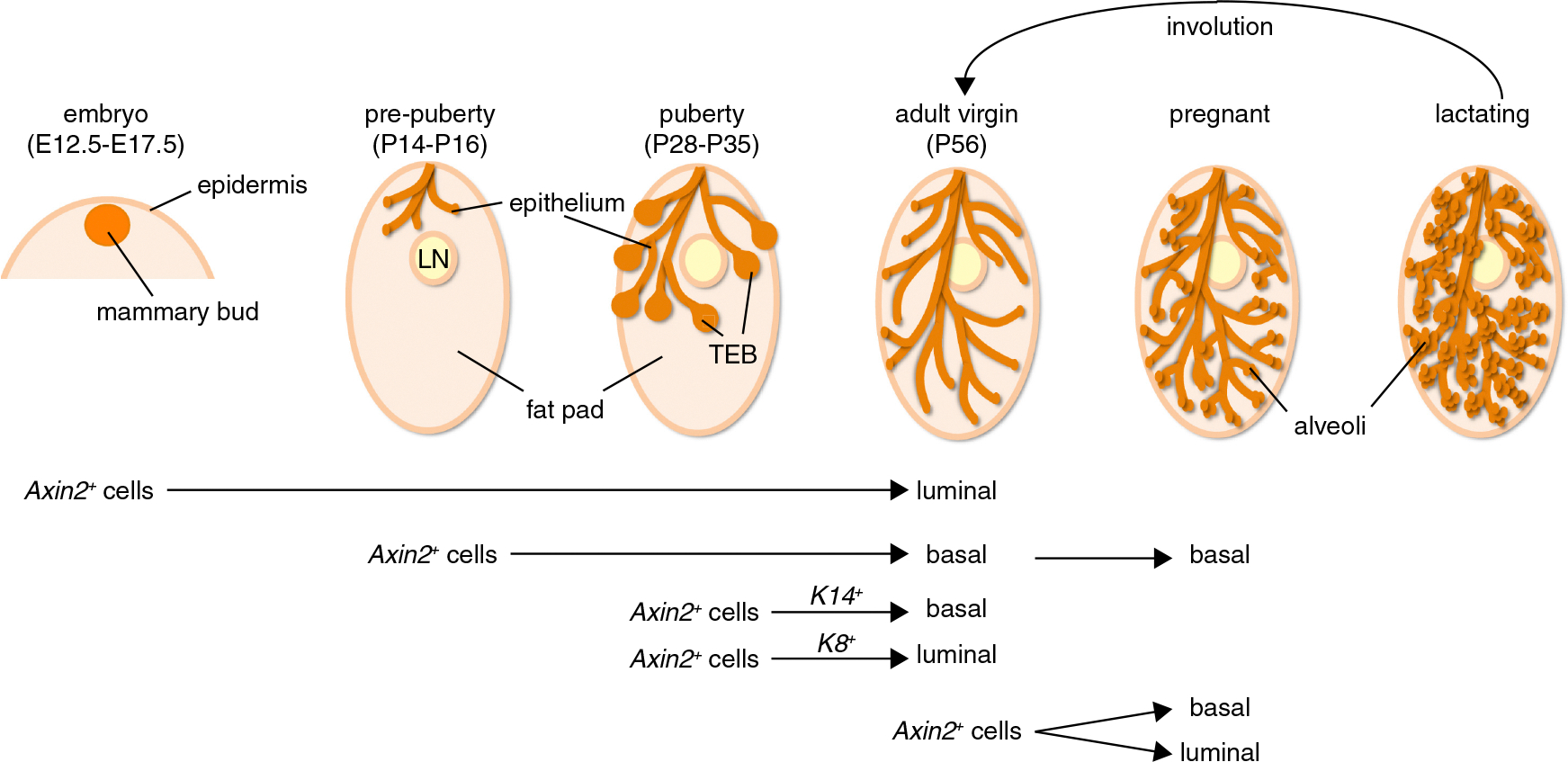
Contribution of Wnt/β-Catenin-Responsive Cells to the Mammary Epithelial Network *Axin2* marks mammary epithelial cells throughout mammogenesis. Our experiments reveal substantial switches in Wnt/β-catenin signaling at distinct developmental stages and dynamic changes in the corresponding cell fate of the *Axin2*^+^ populations. The mammary gland stem and progenitor cell hierarchy might thus hold room for both unipotent (i.e., during puberty) and multipotent (i.e., in the adult) stem cells. See text for details.

First, we observe dynamic changes in *Axin2* expression and the corresponding fate of Wnt/β-catenin-responsive cells depending on the time of TM administration (summarized in Figure 7). Most striking in this regard is the switch in Wnt/β-catenin signaling that occurs around the time of birth. Whereas *Axin2*^+^ cells in the embryo mark the prospective luminal cell lineage (Figure 2), Wnt/β-catenin-responsive cells have become exclusively committed to the basal cell fate in 2-week-old pups (Figure 3).

Although the mammary gland is generally considered to be relatively quiescent in early postnatal life, this observation suggests that important cell fate decisions do in fact occur during this period. For instance, it is at this point that the unipotent, Wnt/β-catenin-responsive myoepithelial stem cells (Figures 3 and 4) are specified. Importantly, the offspring of these prepubescent *Axin2*^+^ cells remain restricted to the basal cell fate during pregnancy, when they expand in number and give rise to large clusters of contractile, myoepithelial cells that surround the alveoli. Finally, these cells survive multiple rounds of pregnancy and involution, demonstrating their potential for long-term self-renewal.

Whereas only the basal lineage is Wnt/β-catenin-responsive prior to the onset of puberty, *Axin2*^+^ cells arise de novo in TEBs, where they mark the prospective luminal lineage (Figure 5). Together, these findings support a model in which two Wnt/β-catenin-responsive lineages arise in consecutive order to give rise to independent basal and luminal cell lineages during puberty. This is in agreement with a recent publication by Van Keymeulen and colleagues, although at present it remains unknown how far the *Axin2*^+^ prepubescent and pubescent cell lineages we identified overlap with the *K14*^+^ and *K8*^+^ lineages marked during puberty by the Cre- and rtTA-transgenic lines used in that study (Van Keymeulen et al., 2011).

### Differences between Transplantation and In Situ Developmental Potential

By directly comparing the behavior of the same cell population in transplantation and lineage tracing experiments, we uncovered important differences between a cell′s regenerative and normal developmental potential. As shown in Figure 4, the prepubescent Wnt/β-catenin-responsive cell lineage is restricted to the basal cell fate throughout puberty and multiple rounds of pregnancy. Yet in spite of this, these cells are fully capable of regenerating both basal and luminal layers upon (serial) transplantation. Interestingly, it was recently demonstrated that basal Lin^−^;CD24^+^;CD29^hi^ cells only adopt a multipotent fate in transplantation assays when they are transplanted in the complete absence or with a limiting amount of luminal Lin^−^;CD24^hi^;CD29^+^ cells (Van Keymeulen et al., 2011).

One explanation for the apparent discrepancy between the developmental and regenerative potential of these cells might be that in a normal developmental context they fulfill a role as facultative stem cells. As such, their multipotent potential might be recruited only as needed, for instance, after tissue damage. However, in light of published reports that even progenitor cells of a completely different origin can be reprogrammed by the microenvironment of the fat pad to adopt a mammary cell fate (Booth et al., 2008; Boulanger et al., 2012), there is also the distinct possibility that transplantation unlocks a regenerative potential that is normally not utilized in vivo. In either case, these results argue that lineage tracing should become the new standard by which to measure normal developmental stem cell potential.

### Does the Bipotent Adult Mammary Stem Cell Exist?

Cumulative work in the field has long suggested a (supra)basal location for the adult mammary stem cell (Chepko and Smith, 1997; Shackleton et al., 2006; Smith et al., 2002). While this cell is assumed to give rise to alveolar structures during pregnancy, its existence has not been formally demonstrated. In fact, recently published lineage tracing experiments suggest that basal and luminal alveolar cells can derive from independent precursors that are set aside during or prior to the onset of puberty (Van Keymeulen et al., 2011). Indeed, our own data demonstrate the specification of a unipotent, Wnt/β-catenin-responsive basal stem cell in prepubescent mice. As a result, the existence of a bipotent adult stem cell remains contested.

The only way to directly probe its existence, however, is to induce recombination in the adult virgin mammary gland and track the fate of these labeled cells through multiple rounds of pregnancy, lactation, and involution. We performed this experiment by administering TM to adult virgin mice and analyzing the contribution of Wnt/β-catenin-responsive cells to the formation of alveoli. Interestingly, we observed clonal GFP^+^ clusters containing adjacent labeled basal and luminal alveolar cells. These cells are long lived and continue to give rise to both basal and luminal offspring in subsequent pregnancies, demonstrating that *Axin2* is a stem cell marker for both the basal and luminal lineage in the adult mammary epithelium. It is a formal possibility that these GFP^+^ basal and luminal alveolar cells arose from independent basal and luminal precursors, in which case *Axin2* would mark the long-sought-after adult luminal stem cell. However, we consider this to be unlikely for a number of reasons.

First, while we are able to detect a tiny fraction of GFP^+^ cells in the luminal cell population by FACS analysis after TM administration to adult virgin mice (Figure 6E), the gated cell populations are not pure and these rare GFP^+^ cells could therefore represent carryover from basal or stromal cell populations. This is supported by the fact that we observe a similar percentage of GFP^+^ cells in the luminal cell population when recombination is induced in prepubescent mice (Figure 3M), yet we never find prepubescent *Axin2*^+^ cells to give rise to luminal alveolar cells in lineage tracing experiments, even after multiple pregnancies.

Second, after administering TM to adult virgin *Axin2*^*CreERT2*/+^; *R26R*^*mTmG*/+^ mice, we detect labeled cells in the basal layer by microscopic analyses. This is in agreement with published data showing enrichment for Wnt/β-catenin pathway receptor components in basal cells (Kendrick et al., 2008), as well as with our earlier observation that *Axin2*^*lacZ*^ is expressed by cells in the basal layer of the adult virgin mammary gland (Zeng and Nusse, 2010).

Finally, given the low frequency of labeled cell clusters during pregnancy, the finding of adjacent GFP^+^ basal and luminal alveolar cells within the same cluster (Figures S5–S7) suggests that these cells are likely to be descendants of a common precursor. Thus, our data support a model in which *Axin2*^*CreERT2*^ marks a bipotent adult stem cell, suggesting that unipotent and multipotent stem cells might coexist in the mammary epithelium. Ruling out that independent basal and luminal adult stem cells are *Axin2*^+^ positive requires further experimentation using combinations of more specific stem cell and lineage markers. In either case, we can definitively conclude that Wnt/β-catenin-responsive cells in the adult virgin mammary epithelium are long-lived stem cells that survive multiple rounds of lobuloalveolar tissue turnover. In future studies, it will be of particular interest to determine the relationship of these *Axin2*^+^ adult mammary stem cells to the K14^+^, K8^+^, and Lgr5^+^ cell populations labeled during puberty (Van Keymeulen et al., 2011; Visvader and Lindeman, 2011) as well as to the previously described self-renewing population of parity-identified mammary epithelial cells (Boulanger et al., 2005; Wagner et al., 2002).

## EXPERIMENTAL PROCEDURES

### Animals

To generate mice expressing *Cre*^*ERT2*^ under control of the endogenous *Axin2* promoter and enhancer sequences, we modified the original targeting construct used to generate *Axin2*^*lacZ*^ mice (Lustig et al., 2002). See Supplemental Experimental Procedures for details.

*Rosa26-lacZ* (*R26R*^*lacZ*^; stock 3474) and *Rosa26-mTmG* (*R26R*^*mTmG*^; stock 7676) reporter mice were obtained from Jackson Laboratories. *Axin2*^*lacZ*^ mice were a gift from Dr. W. Birchmeier. Nude mice (*Nu/Nu*; stock 088) were obtained from Charles River. All experiments were approved by the Stanford University Animal Care and Use Committee and performed according to NIH guidelines.

### Labeling and Tracing Experiments

Unless otherwise indicated, mice received a single intraperitoneal injection of a 10–20 mg/ml stock solution of tamoxifen (TM) in corn oil/10% ethanol, totaling 4 mg/25 g body weight. This corresponds to a total dose of 1 mg TM for prepubescent mice (injected between P14 and P16), 2 mg TM for pubescent mice (injected between P28 and P35), and 4 mg TM for adult virgins (injected between P56 and P63). To induce sporadic recombination in embryos, we gave a single injection of TM to pregnant mothers between E12.5 and E17.5, totaling 0.5 mg/25 g body weight.

### Immunofluorescence and Immunohistochemistry

See Supplemental Experimental Procedures for details on the whole-mount analyses of fluorescent and X-gal-stained mammary glands, as well as for details on the X-gal staining procedure.

PFA-fixed or ethanol-fixed samples were embedded in paraffin according to standard techniques. Tissues were sectioned on a Leica Microtome. Individual sections (2–5 μm for X-gal-stained samples, 10 μm for *R26R*^*mTmG*^ samples) were mounted onto superfrost slides and dried overnight at 37°C. For paraffin fixed samples, antigen retrieval was performed in Tris/EDTA (pH 9.0). Sections were stained with antibodies recognizing keratin 14 (K14, 1:500, Covance), smooth muscle actin (SMA, 1:1,000, Sigma), keratin 8 (K8, 1:250, Troma-1, DSHB), laminin (1:250, Sigma), and GFP (1:1,000, Abcam). They were processed for immunofluorescence (with Alexa 488- or Cy3- or Cy5-conjugated secondary antibodies, Jackson Immunoresearch) or immunohistochemistry (with the Vectastain ABC system and DAB substrate, Vector Laboratories) according to the manufacturer’s instructions. The dTomato signal was lost when tissues were processed for routine paraffin embedding and histology, enabling us to use both the Cy3 and the Cy5 channel for the detection of fluorescently labeled antibodies against the different markers.

### Flow Cytometry and Cleared Fat Pad Transplantation

Mammary epithelial cells were stained with a cocktail of antibodies directed against Ter119, CD31, CD45, CD24, CD29, and CD49f, followed by analysis and sorting on a BD Facs Aria (Beckton Dickinson). To test their mammary repopulating behavior, we injected sorted GFP^+^ Lin^−^;CD24^+^;CD29^hi^ cells into the cleared fat pad of 21-day-old nude recipient mice. Outgrowths were analyzed 4–8 weeks after transplantation. MRU frequencies were calculated using L-calc software (Stem Cell Technologies). See Supplemental Experimental Procedures for details on the cell isolation, staining, and transplantation procedures.

## Supplementary Material

supplement

SUPPLEMENTAL INFORMATION

Supplemental information for this article includes seven figures, one table, and Supplemental Experimental Procedures and can be found with this article online at http://dx.doi.org/10.1016/j.stem.2012.05.023.
