# Supplementary material for: Developmental Stage and Time Dictate the Fate of Wnt/β-Catenin-Responsive Stem Cells in the Mammary Gland
Source: Cell Stem Cell. Author manuscript; Available in PMC 2026 May 8. (PMC13155203; doi:10.1016/j.stem.2012.05.023)
Supplement: supplement [file NIHMS2156153-supplement-supplement.pdf]

Cell Stem Cell, Volume *11*

Supplemental Information

Developmental Stage and Time Dictate the Fate of Wnt/β-Catenin-Responsive Stem Cells in the Mammary Gland

Renée van Amerongen, Angela N. Bowman, and Roel Nusse

Supplemental Inventory

Supplemental Data

- Figure S1. *Axin2*^{CreERT2} marks intestinal stem cells (related to Figure 1)
- Figure S2. FACS strategy for detecting GFP^{+} mammary epithelial cells in adult *Axin2*$^{CreERT2/+}$;*R26R*$^{mTmG/+}$ mice (related to Figures 2, 3 and 6)
- Figure S3. Rare Wnt/β-catenin-responsive cells in the prepubescent mammary epithelium are restricted to the basal cell fate (related to Figure 3)
- Figure S4. Cells marked by *Axin2*^{CreERT2} in the adult virgin mammary gland display regenerative potential in a transplantation assay (related to Figure 6)
- Figure S5. Wnt/β-catenin-responsive cells in the adult virgin form alveoli during pregnancy
(related to Figure 6)
- Figure S6. Wnt/β-catenin-responsive cells in the adult virgin can give rise to adjacent basal and luminal alveolar cells during pregnancy (related to Figure 6)
- Figure S7. Wnt/β-catenin-responsive cells in the adult virgin are long-lived stem cells that contribute to basal and luminal alveolar cells during multiple rounds of pregnancy (related to Figure 6)
- Table S1. Overview of transplantation assays (related to Figure 4 and Figure 6)

Supplemental Experimental Procedures

- Generation of *Axin2*^{CreERT2} mice
- Details on whole-mount confocal microscopy
- Details on whole-mount X-gal staining procedure
- Details on mammary epithelial cell isolation, flow cytometry and cleared fat pad transplantation.

Supplemental References

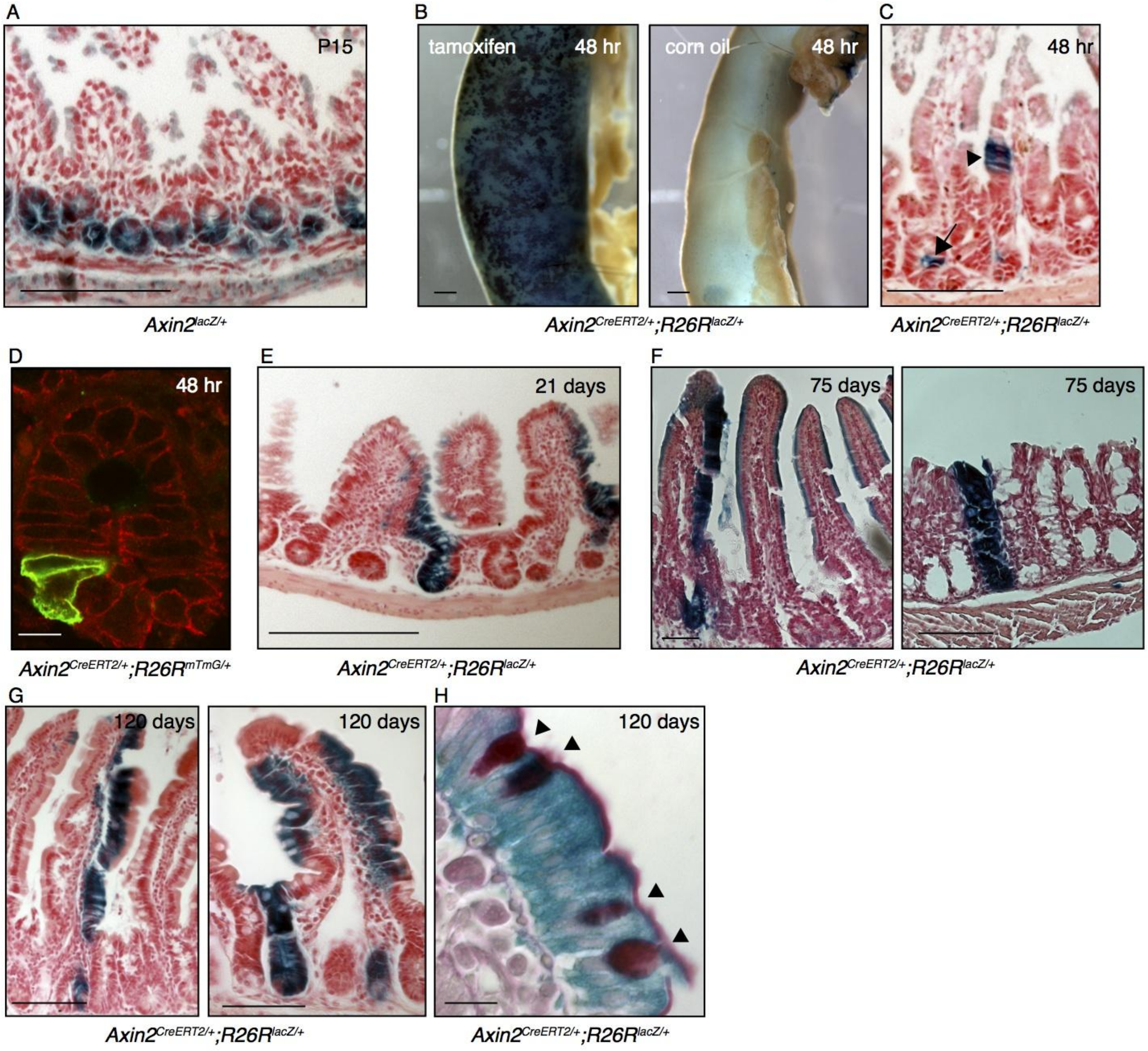

Figure S1. ***Axin2***$^{\mathbf{\mathit{CreERT2}}}$ **marks intestinal stem cells (related to Figure 1)**

(A) Tissue section of X-gal stained intestine from *Axin2*^{lacZ} mice at P15, demonstrating that Wnt/β-catenin signaling is restricted to the lower half of the intestinal crypt, which is where the intestinal stem cells reside. Scale bar is 100 μm. (B) Whole-mount X-gal staining of the intestine from *Axin2*$^{CreERT2/+}$;*R26R*$^{lacZ/+}$ mice, demonstrating efficient labeling of intestinal crypts 48 hours after receiving a single dose of tamoxifen (1 mg/25 grams, left) at P21 and

showing no recombination in *Axin2*$^{CreERT2/+}$;*R26R*$^{lacZ/+}$ littermates that received corn oil instead of tamoxifen (right). Scale bar is 1 mm. (C) Tissue section of X-gal stained intestines from *Axin2*$^{CreERT2/+}$;*R26R*$^{lacZ/+}$ mice, showing small patches of labeled cells along the villus 48 hours after switching (arrowheads). These patches represent transiently amplifying cells that were labeled 48 hours prior and which have since stopped dividing. Labeled cells are also present in the crypts (arrow) as either single cells or small patches. Scale bar is 100 μm. (D) Confocal microscopy, showing that identical results are obtained with adult *Axin2*$^{CreERT2/+}$;*R26R*$^{mTmG/+}$ mice. Sporadic recombination (green) can be detected in otherwise unrecombined (red) crypts 48 hours after the administration of 0.2 mg/25 grams tamoxifen. Scale bar is 10 μm. (E) Tissue section of the whole-mount preparation shown in Figure 1C', showing the X-gal stained intestine from an *Axin2*$^{CreERT2/+}$;*R26R*$^{lacZ/+}$ mouse that received tamoxifen at P21 and demonstrating the labeling of entire crypt/villus structures 21 days after recombination. (F-G) Tissue sections of X-gal stained intestine from *Axin2*$^{CreERT2/+}$;*R26R*$^{lacZ/+}$ mice that were traced for 75 (F) or 120 (G) days, showing persistent labeling in the proximal (F, left and G, left, scale bars are 100 μm (F) and 200 μm (G)) and distal (G, right, scale bar is 50 μm) small intestine as well as the colon (F, right, scale bar is 100 μm). These labeled crypt/villus structures are long-lived and persist in spite of the fact that the intestinal epithelium has turned over multiple times, offering definitive proof that *Axin2*^{CreERT2} has marked intestinal stem cells. (H) Tissue section of X-gal and Periodic Acid-Schiff (PAS) stained intestine from *Axin2*$^{CreERT2/+}$;*R26R*$^{lacZ/+}$ mice , demonstrating that labeled cells continue to contribute to differentiated cells in the villus, including goblet cells (arrow heads), at 120 days post tamoxifen administration. Scale bar 20 is μm.

van Amerongen et al., Supplementary Figure S2

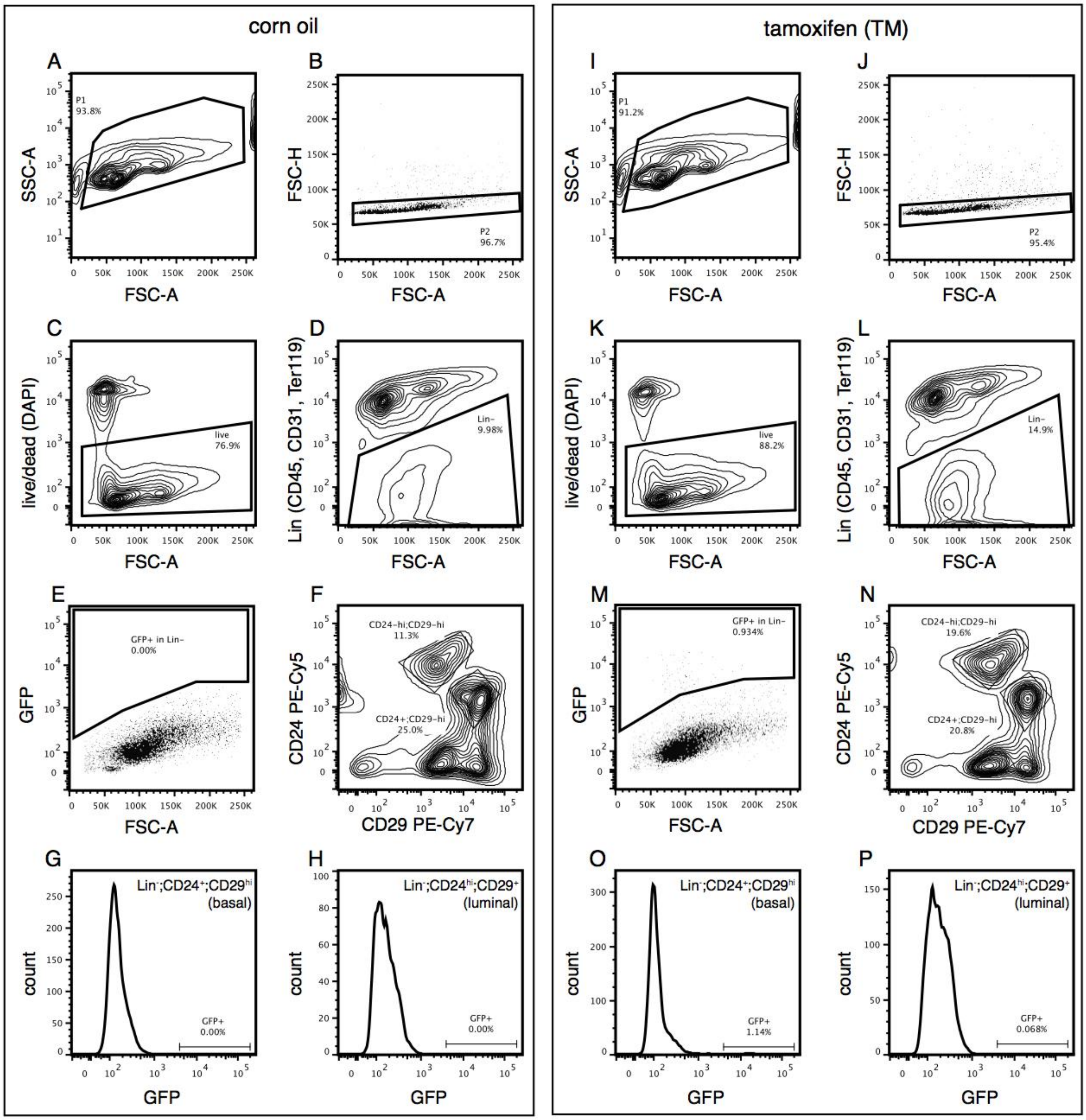

Figure S2. FACS strategy for detecting GFP^{+} mammary epithelial cells in adult *Axin2$^{CreERT2/+}$*;*R26R$^{mTmG/+}$* mice (related to Figures 2, 3, and 6)

FACS strategy used for the analyses in Figures 2J-2M, 3L-3O and 6D-6G, as well as for the sorting of GFP^{+} cells for the transplantation assays summarized in Table S1. This

particular figure illustrates the procedure on adult virgin *Axin2*$^{CreERT2/+}$;*R26R*$^{mTmG/+}$ mice that were injected with corn oil (A-H) or tamoxifen (4mg/25 grams body weight, I-P) and analyzed 48 hours later. In our hands, this is the earliest timepoint at which labeling can be detected in the mammary gland using the *R26R*^{mTmG} reporter.

Basal and luminal mammary epithelial cell populations can be distinguished by flow cytometry based on their differential expression of CD24 and CD29 (Shackleton et al., 2006; Sleeman et al., 2006). Single cell suspensions were prepared from mammary glands and analyzed by flow cytometry. (A,I) Cell debris was excluded from the analysis by gating on P1. (B,J) Doublets were discarded by gating on P2. (C,K) Dead cells were excluded based on their uptake of DAPI by gating only live cells. (D,L) Hematopoietic Lin^{+} cells were excluded based on their expression of CD31, CD45 and/or Ter119. Only Lin$^{—}$ cells were used for the remainder of the analysis. (E,M) No GFP^{+} Lin$^{—}$ cells were detected in mice that had been injected with corn oil, but a small percentage of GFP^{+} Lin$^{—}$ cells could be detected in mice that had received tamoxifen. (F,N) CD24 and CD29 expression in Lin$^{—}$ cells to identify the different mammary epithelial cell populations. Basal cells are Lin$^{—}$;CD24^{+};CD29^{hi}. This population is enriched for cells with regenerative potential (the so-called MRUs), as measured by transplantation into the cleared fat pad. For transplantation experiments, GFP^{+} cells were sorted from this population. In contrast, the Lin$^{—}$;CD24^{hi};CD29^{+} population contains luminal progenitors and differentiated luminal cells. (G,O) After 48 hours GFP^{+} cells are detected in the basal Lin$^{—}$;CD24^{+};CD29^{hi} population of mice treated with tamoxifen, but not in that of mice treated with corn oil. (H,P) Labeled luminal Lin$^{—}$;CD24^{hi};CD29^{+} cells are rare in tamoxifen treated mice.

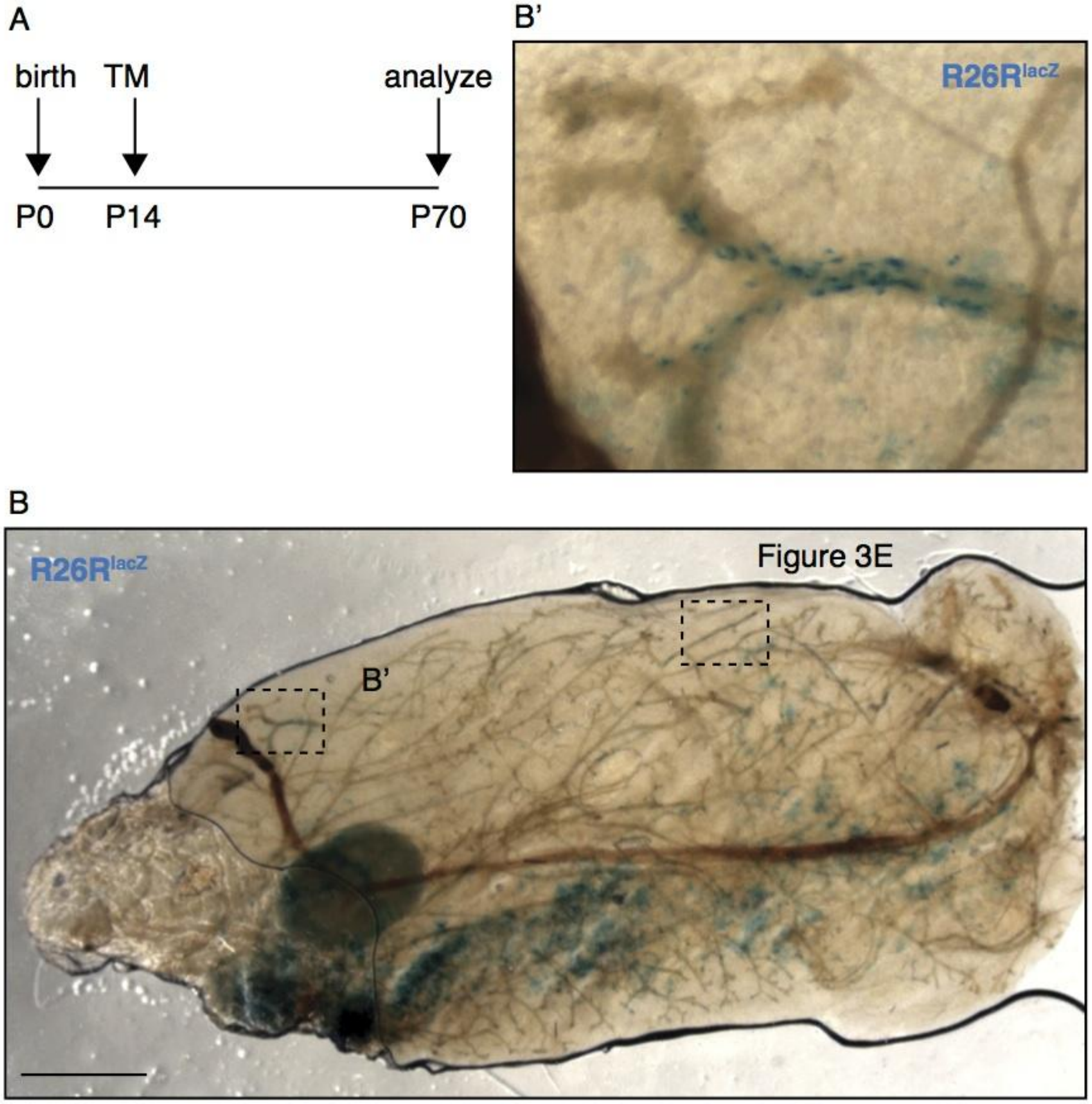

van Amerongen et al., Supplementary Figure S3

Figure S3. Rare Wnt/β-catenin-responsive cells in the prepubescent mammary epithelium are restricted to the basal cell fate (related to Figure 3)

(A) Schematic depicting the treatment schedule for the experiment depicted in panel B. To test the fate of Wnt/β-catenin-responsive cells in the prepubescent mammary epithelium, two-week old (P14) *Axin2*$^{CreERT2/+}$;*R26R*$^{lacZ/+}$ pups were injected with tamoxifen (TM) to induce recombination. After a trace period of 8 weeks, mice were analyzed as 10-week old (P70) adult virgins. (B) Whole-mount preparation of an X-gal stained mammary gland from

an adult virgin *Axin2*$^{CreERT2/+}$;*R26R*$^{lacZ/+}$ mouse in which recombination was induced at P14, showing tracts of switched cells that are deposited along the ducts, including the most distal tips. Scale bar is 2 mm. (B’) is a close-up of the corresponding region boxed in (B). A close-up of the other boxed region is depicted in Figure 3E.

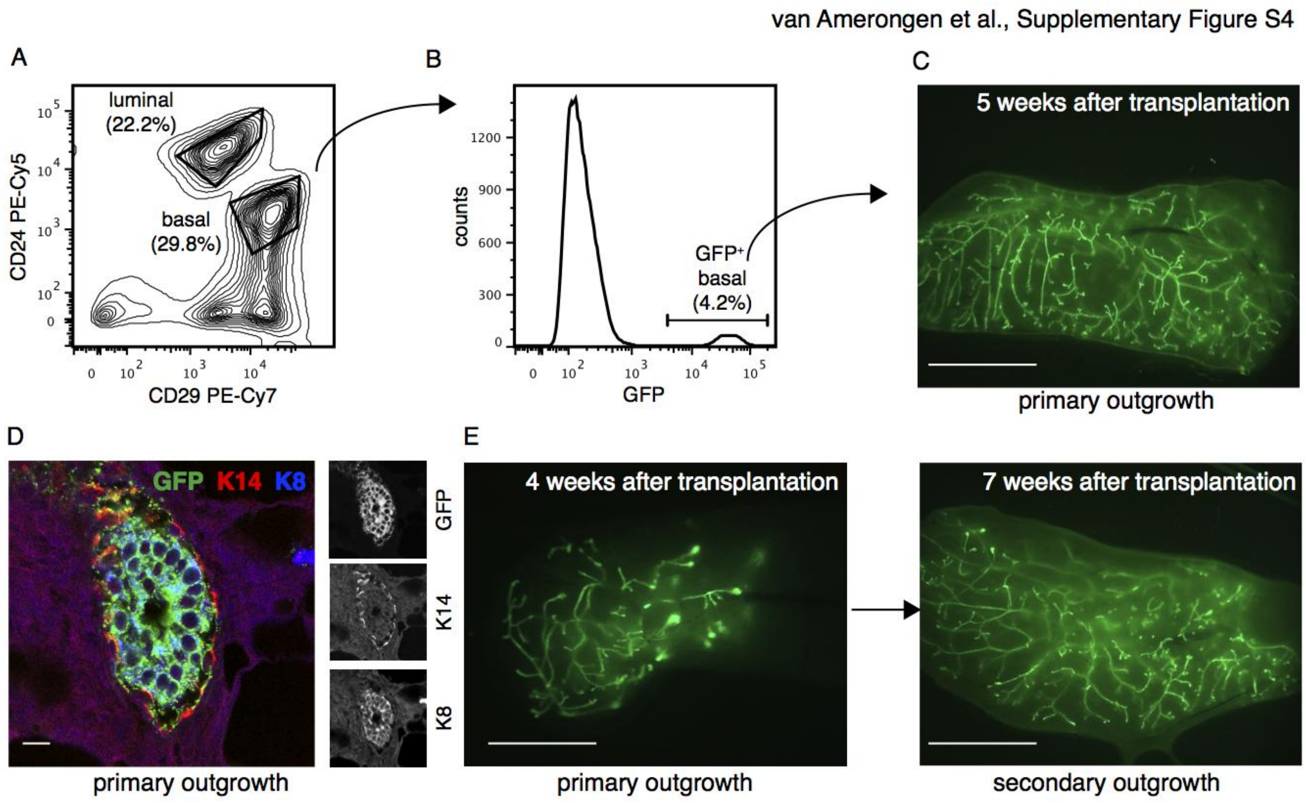

Figure S4. Cells marked by *Axin2*^{CreERT2} in the adult virgin mammary gland display regenerative potential in a transplantation assay (related to Figure 6)

(A,B) FACS analysis demonstrating the procedure for isolating GFP^{+} Lin^{-};CD24^{+};CD29^{hi} cells from *Axin2*$^{CreERT2/+}$;*R26R*$^{mTmG/+}$ mice. (A) Distribution of basal and luminal populations based on CD24 and CD29 expression in Lin^{-} mammary cells from adult virgin *Axin2*$^{CreERT2/+}$;*R26R*$^{mTmG/+}$ mice that received tamoxifen at 8 to 10 weeks of age. (B) GFP^{+} cells were sorted from the gated Lin^{-};CD24^{+};CD29^{hi} population (see Supplementary Figure S2 for the FACS strategy) and anywhere from 50 to 1000 cells were injected into the cleared fat pad of 21 day old recipients.

(C) Whole-mount image of a mammary transplant analyzed 5 weeks after transplantation of 50 GFP^{+} Lin^{-};CD24^{+};CD29^{hi} cells. (D) Immunostaining of a histological section from a GFP^{+} outgrowth generated following the transplantation of GFP^{+} Lin^{-};CD24^{+};CD29^{hi} cells

isolated from *Axin2*$^{CreERT2/+}$;*R26R*$^{mTmG/+}$ mice that received tamoxifen as adults. The regenerated ductal tree contains both GFP^{+} basal (K14^{+}) and luminal (K8^{+}) layers. (E) Whole-mount image of a primary outgrowth derived from the transplantation of 500 GFP^{+} Lin$^{—}$;CD24^{+};CD29^{hi} cells isolated from *Axin2*$^{CreERT2/+}$;*R26R*$^{mTmG/+}$ mice that were received tamoxifen as adults (left) and its corresponding secondary outgrowth (right) derived from the transplantation of 1500 GFP^{+} Lin$^{—}$;CD24^{+};CD29^{hi} cells isolated from the primary outgrowth. Scale bars are 10 μm in (D) and 500 μm in (C,E). (See also Table S1.)

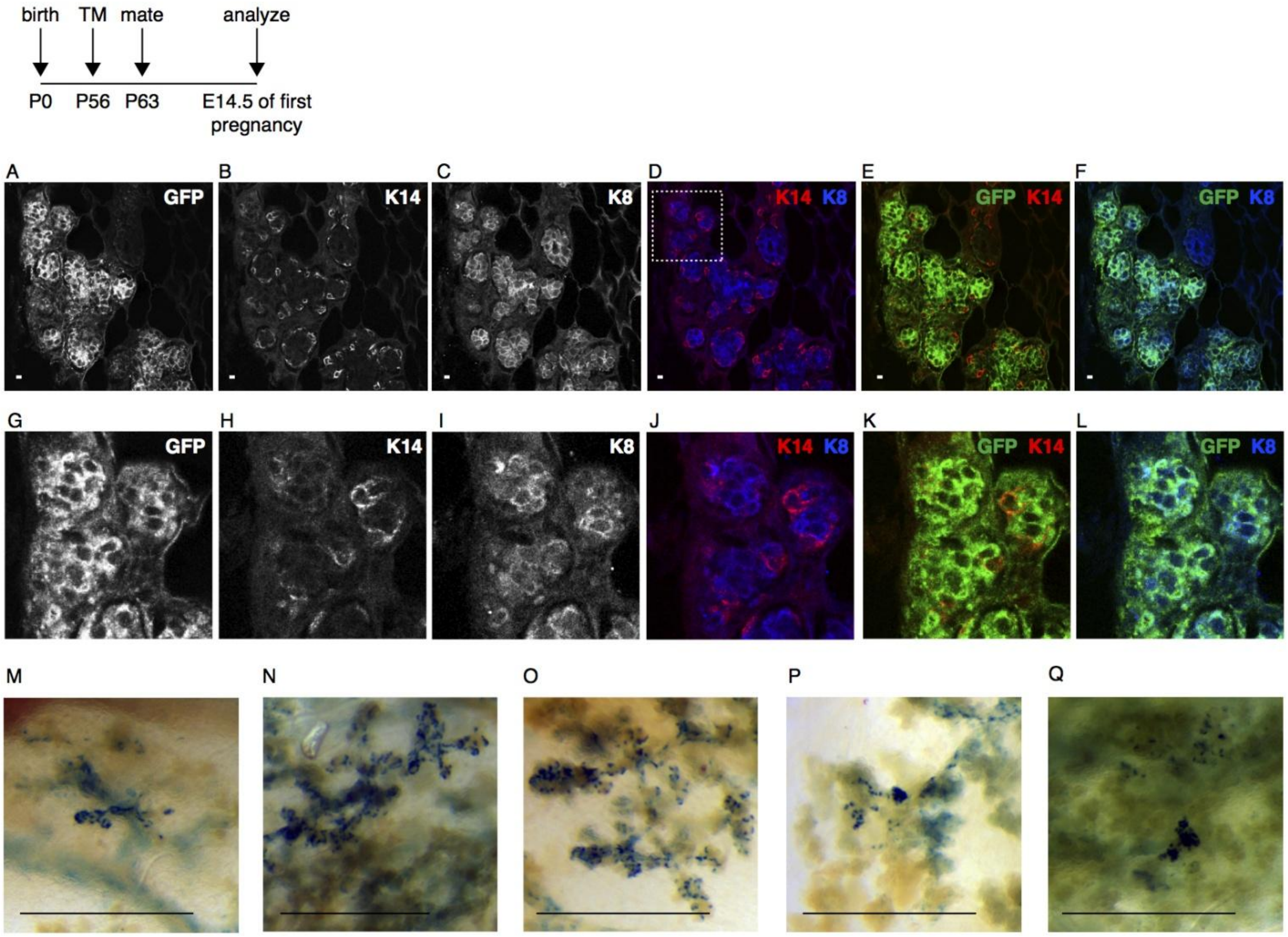

Figure S5. Wnt/β-catenin-responsive cells in the adult virgin form alveoli during pregnancy (related to Figure 6)

(A-F) Immunostaining for GFP (A,E,F), K14 (B,D,E) and K8 (C,D,F) on a mammary gland from an *Axin2*$^{CreERT2/+}$;*R26R*$^{mTmG/+}$ mouse harvested at day 14.5 of pregnancy (treatment schedule similar to that in Figure 6H, depicted at the top), showing an alveolar cluster made up out of GFP^{+} cells. Scale bars are 10 μm.

(G-L) Magnification of the area boxed in (D) shows that GFP^{+} cells (G,K,L) contribute to both the K14^{+} basal layer (H,J,K) as well as in the K8^{+} luminal layer (I,J,L).

(K-L) Adjacent GFP^{+} basal and luminal alveolar cells can be detected.

(M-Q) Whole-mount preparations of X-gal stained mammary glands from

Axin2$^{CreERT2/+}$;*R26R*$^{lacZ/+}$ mice that received tamoxifen as adult virgins, showing similar clusters of labeled cells at day 8 (M), 16 (N) or day 18 (O-Q) of gestation. Scale bars are 500 μm.

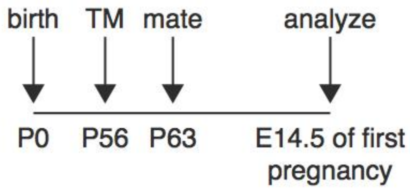
birth
TM
mate
analyze
P0
P56
P63
E14.5 of first pregnancy

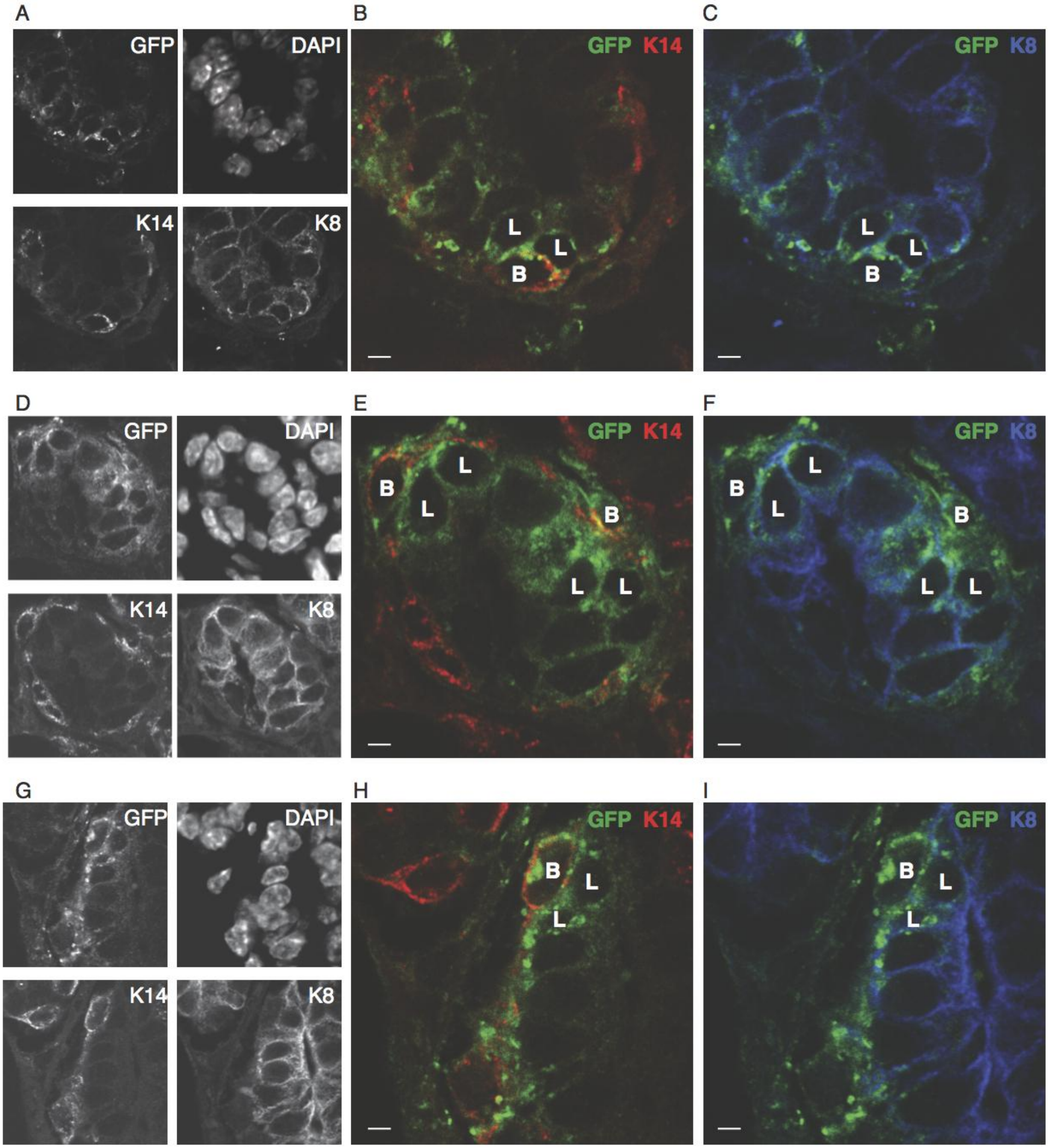
A
GFP
DAPI
K14
K8
B
GFP K14
L
L
B
C
GFP K8
L
L
B
D
GFP
DAPI
K14
K8
E
GFP K14
B
L
L
B
L
L
F
GFP K8
B
L
L
B
L
L
G
GFP
DAPI
K14
K8
H
GFP K14
B
L
L
I
GFP K8
B
L
L

Figure S6. Wnt/β-catenin-responsive cells in the adult virgin can give rise to adjacent basal and luminal alveolar cells during pregnancy (related to Figure 6)
Confocal microscopy on immunostained tissue sections of the mammary gland from a pregnant *Axin2*$^{CreERT2/+}$;*R26R*$^{mTmG/+}$ mouse after tamoxifen administration as an adult virgin (treatment schedule depicted at the top, similar to that in 6H). High power magnification demonstrates the presence of adjacent GFP^{+} basal and luminal alveolar cells. (A,D,G) Single channel images showing the GFP, K14 and K8 signal. DAPI shows cell nuclei for orientation. (B,E,H) Two-color overlay showing the presence of basal, K14^{+} GFP^{+} cells next to K14$^{—}$ GFP^{+} luminal cells. (C,F,I) Complementary overlay showing the presence of luminal, K8^{+} GFP^{+} cells next to K8$^{—}$ GFP^{+} basal cells. Scale bars are 1 μm. B = basal. L = luminal.

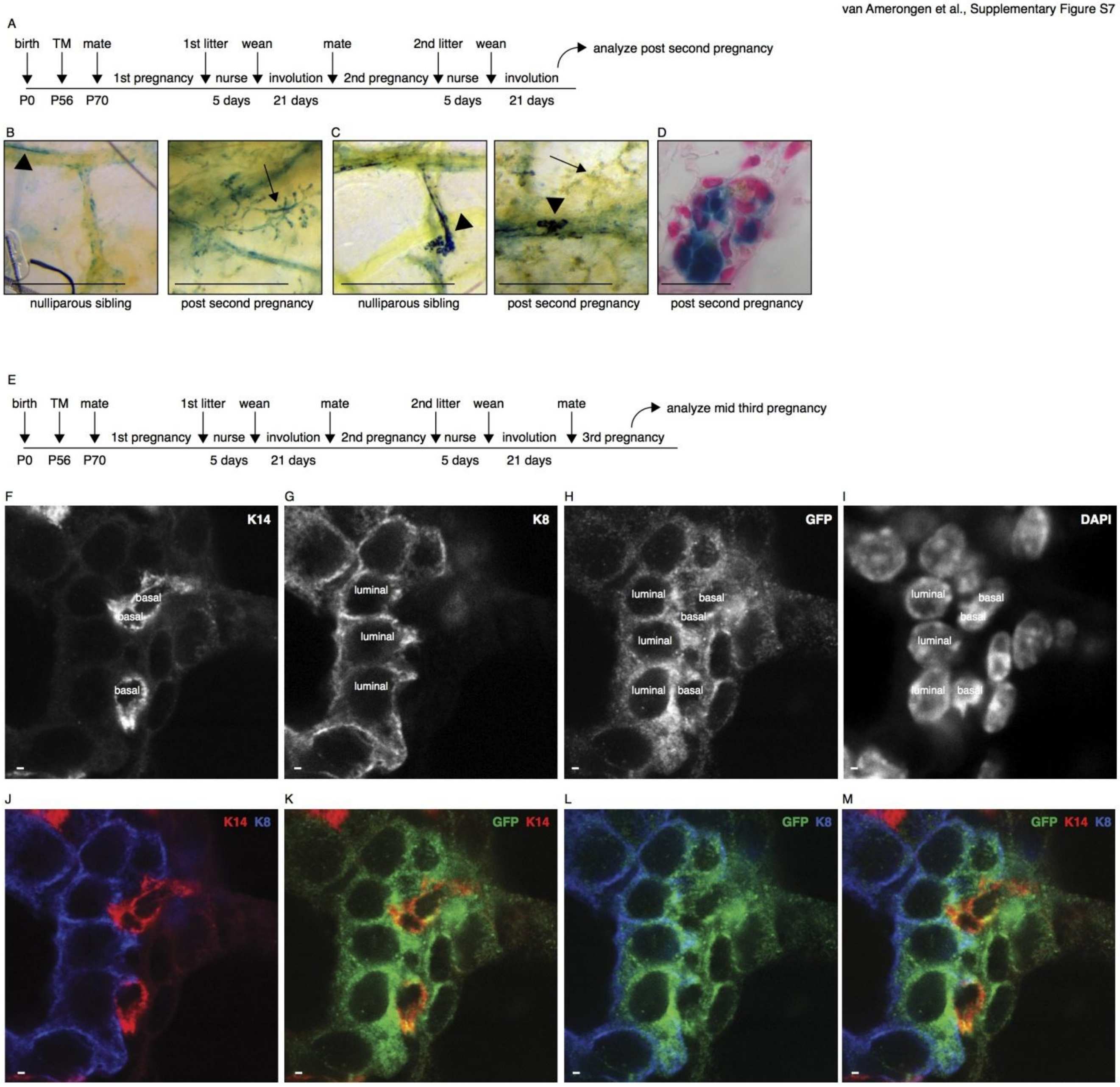

Figure S7. Wnt/β-catenin-responsive cells in the adult virgin are long-lived stem cells that contribute to basal and luminal alveolar cells during multiple rounds of pregnancy (related to Figure 6)

(A) Treatment schedule for analyzing the contribution of Wnt/β-catenin-responsive cells to alveolar structures during multiple rounds of pregnancy. Adult virgin *Axin2*$^{CreERT2/+}$;*R26R*$^{lacZ/+}$ mice received a single dose of tamoxifen (TM) at P56. They were mated at P70 and monitored for signs of pregnancy. Once pups were born, mice were

allowed to nurse to ensure complete terminal differentiation of the mammary gland. After forced weaning of the pups on postnatal day 5, the mice were housed for 21 days to allow complete involution of the mammary epithelium. At this point, mice were re-mated and submitted to up to two additional cycles of pregnancy, lactation and involution according to an identical schedule. Nulliparous siblings received tamoxifen simultaneously but were never mated, and were analyzed together with their multiparous siblings as controls. (B-C) Whole-mount preparations of X-gal stained mammary glands from the experiment outlined in (A), depicting nulliparous $Axin2^{CreERT2/+}$;$R26R^{lacZ/+}$ mice (left) and their multiparous siblings after two rounds of pregnancy (right), and demonstrating that $lacZ^{+}$ cells are still present regardless of parity status approximately 15-16 weeks after switching. Scale bars are 500 μm. (D) Histological tissue section of an X-gal stained $Axin2^{CreERT2/+}$;$R26R^{lacZ/+}$ mammary gland, showing a close-up of a regressing alveolus at 21 days of involution after the second pregnancy. Labeled cells are still present. Scale bar is 20 μm.

(E) Treatment schedule for analyzing the contribution of Wnt/β-catenin-responsive cells to alveolar structures during multiple rounds of pregnancy as used for panels (F-M). The experimental set up is the same as for panel (A), with the exception that mammary glands were analyzed during the third pregnancy at midgestation. (F-M) High power magnification and confocal microscopy of the alveolar structure depicted in Figure 6O. (F-I) Single channel images depicting immunostaining for the basal marker K14 (F), the luminal marker K8 (G) and GFP (H). DAPI shows cell nuclei for orientation (I). Luminal and basal cells are indicated in each panel. (J-L) Two-color overlay showing basal and luminal cells (J) and adjacent GFP^{+} basal (K) and GFP^{+} luminal (L) cells. (M) Three-color overlay showing adjacent GFP^{+} $K14^{+}$ basal and GFP^{+} $K8^{+}$ luminal cells.

Table S1. Overview of transplantation assays (related to Figure 4 and Figure 6)

**Cells isolated from adult *Axin2*$^{CreERT2/+}$;*R26R*$^{mTmG/+}$ mice labeled prepuberty at P14-P16 (depicted in Figures 5H and 5I)**		
	Number of cells injected	Number of primary outgrowths
GFP$^{—}$ Lin$^{—}$;CD24^{+};CD29^{hi} (red outgrowths - control)	2500	2/2
	1250	2/2
	625	2/2
	500	2/2
	312	0/2
	156	0/2
GFP^{+} Lin$^{—}$;CD24^{+};CD29^{hi} (green outgrowths)	500	7/7
	250	6/10
	140	6/10
	50	2/10

**Cells isolated from adult labeled *Axin2*$^{CreERT2/+}$;*R26R*$^{mTmG/+}$ mice labeled at P56 (depicted in Figure S5)**		
	Number of cells injected	Number of primary outgrowths
GFP^{+} Lin$^{—}$;CD24^{+};CD29^{hi} (green outgrowths)	1000	5/5
	500	5/14
	300	6/9
	250	6/9
	200	3/5
	100	0/10
	50	7/8

To obtain enough GFP^{+} cells, we pooled 4 to 6 mammary glands (from 2 to 3 adult virgin mice) for each sort. This allowed us to isolate between 1000 and 2000 GFP^{+} Lin$^{—}$;CD24^{+};CD29^{hi} cells per experiment.

Calculated MRU (mammary repopulating unit) frequencies:
1/192 MRUs for the prepuberty labeled cells (95% confidence interval 1/120 – 1/307)
1/374 MRUs for the adult virgin labeled cells (95% confidence interval 1/254 – 1/550)

Supplemental Experimental Procedures

Generation of ***Axin2^{CreERT2}*** mice

To generate mice expressing *Cre^{ERT2}* under control of the endogenous *Axin2* promoter and enhancer sequences, we modified the original targeting construct used to generate *Axin2^{lacZ}* mice (Lustig et al., 2002). We replaced the *lacZ* gene (knocked into exon 2) with *Cre^{ERT2}* at the exact same nucleotide position according to standard cloning techniques. The targeting construct was linearized with NotI and electroporated into R1 embryonic stem (ES) cells. Following alkaline transfer of EcoRI digested genomic DNA onto a Hybond XL membrane (GE Healthcare), ES cell clones that had undergone homologous recombination were identified by Southern blot analysis using Rapid-Hyb buffer (GE Healthcare) and a 5' external probe, which was labeled by random primed labeling using the Rediprime II Labeling System (GE Healthcare) according to the manufacturer's protocol. These clones were expanded and used for blastocyst injections. All experiments in this paper were performed with mice derived from clone 3D3.

Details on whole-mount confocal microscopy

Mammary glands were fixed in 100% ethanol, cleared in methylsalicylate and imaged on a Leica SP2 confocal with a 40x oil objective, allowing detection of both the endogenous GFP and dTomato signals.

Details on whole-mount X-gal staining procedure

X-gal staining was performed according to Hogan (Hogan, 1994) with minor modifications. Mammary glands were fixed in PBS with 0.2% glutaraldehyde, 5 mM EGTA (pH 8.0) and 2 mM $MgCl_2$. Tissues were washed in detergent rinse (PBS with 2 mM $MgCl_2$, 0.01% sodium deoxycholate and 0.02% NP-40) and stained in staining solution (PBS with 2 mM $MgCl_2$, 0.01% sodium deoxycholate, 0.02% NP-40, 5 mM potassium ferricyanide, 5 mM potassium ferrocyanide and 1 mg/ml X-gal) in the dark at room temperature overnight. Following staining, tissues were washed, post-fixed in 4% PFA and processed for paraffin embedding. Whole mounts were photographed under a dissecting scope (Leica) after clearing in orange terpene.

Details on mammary epithelial cell isolation, flow cytometry, and cleared fat pad transplantation

Briefly, mammary cell suspensions were prepared by incubating finely chopped mammary glands in RPMI/5%FBS/1%PSQ/25mM HEPES supplemented with 300 units collagenase 3 (Worthington) per milliliter digestion mix while shaking at 37 ºC for 2 hours. Digestion mixes were washed with DMEM/10%FBS, incubated with red blood cell lysis buffer (Sigma) and spun down in PBS. Cells were incubated with 0.05%Trypsin/EDTA for 5 minutes at 37 ºC.

Cell clumps that formed due to the release of DNA were removed through careful resuspension by pipetting following the addition of serumfree DMEM with 1 ug/ml DNAse I. Cells were incubated for an additional 5 minutes at 37 ºC, after which they were again

carefully resuspended. Digestion was stopped by adding DMEM/10%FBS. Cells were filtered through a 40 μm filter and washed in HBSS/10%FBS.

Between $8x10^6$ and $1x10^7$ mammary epithelial cells were stained in a total volume of 1 ml HBSS/10%FBS supplemented with biotin-conjugated anti-Ter119, anti-CD31 and anti-CD45 (all 1:200, eBioscience) as well as with CD24-PE-Cy5 (1:50, eBioscience), CD29-PECy7 (1:100, eBioscience) and CD49f-APC (1:100, eBioscience) on ice for 30 minutes. Cells were washed in HBSS/10%FBS, spun down for 5 minutes at 1000 rpm and incubated with Streptavidin-eFluor450 secondary antibody (1:1000, eBioscience) and DAPI on ice for 30 minutes. Following an additional wash in HBSS/10%FBS, cells were refiltered through a 40 μm filter and analyzed and sorted on a BD Facs Aria (Beckton Dickinson).

To test their mammary repopulating behavior, GFP^+ $Lin^-CD24^+;CD29^{hi}$ cells were sorted into HBSS/10%FBS, spun down and resuspended at the appropriate concentration in a 1:1 mixture of HBSS/10%FBS and matrigel. Twenty-one day old nude recipient mice were anesthesized by intraperitoneal injection of ketamine/xylazine and 10 μl volumes of GFP^+ $Lin^-CD24^+;CD29^{hi}$ cells were injected into the cleared fat pad.

Supplemental References

Hogan, B. (1994). Manipulating the Mouse Embryo: A Laboratory Manual, Second Edition (Cold Spring Harbor, N.Y.: Cold Spring Harbor Laboratory Press).
Shackleton, M., Vaillant, F., Simpson, K.J., Stingl, J., Smyth, G.K., Asselin-Labat, M.L., Wu, L., Lindeman, G.J., and Visvader, J.E. (2006). Generation of a functional mammary gland from a single stem cell. Nature *439*, 84-88.
Sleeman, K.E., Kendrick, H., Ashworth, A., Isacke, C.M., and Smalley, M.J. (2006). CD24 staining of mouse mammary gland cells defines luminal epithelial, myoepithelial/basal and non-epithelial cells. Breast Cancer Res *8*, R7.
Van Keymeulen, A., Rocha, A.S., Ousset, M., Beck, B., Bouvencourt, G., Rock, J., Sharma, N., Dekoninck, S., and Blanpain, C. (2011). Distinct stem cells contribute to mammary gland development and maintenance. Nature.
Zeng, Y.A., and Nusse, R. (2010). Wnt proteins are self-renewal factors for mammary stem cells and promote their long-term expansion in culture. Cell Stem Cell *6*, 568-577.
